# Ion and Molecular Transport in Solid Electrolytes Studied by NMR

**DOI:** 10.3390/ijms23095011

**Published:** 2022-04-30

**Authors:** Vitaly I. Volkov, Alexander V. Chernyak, Nikita A. Slesarenko, Irina A. Avilova

**Affiliations:** 1 Institute of Problems of Chemical Physics RAS, 142432 Chernogolovka, Russia; sasha_cherniak@mail.ru (A.V.C.); wownik007@mail.ru (N.A.S.); irkaavka@gmail.com (I.A.A.); 2 Scientific Center in Chernogolovka RAS, 142432 Chernogolovka, Russia

**Keywords:** ion-exchange membranes, solid-state electrolytes, NMR spectroscopy, pulsed field gradient NMR, NMR relaxation

## Abstract

NMR is the method of choice for molecular and ionic structures and dynamics investigations. The present review is devoted to solvation and mobilities in solid electrolytes, such as ion-exchange membranes and composite materials, based on cesium acid sulfates and phosphates. The applications of high-resolution NMR, solid-state NMR, NMR relaxation, and pulsed field gradient ^1^H, ^7^Li, ^13^C, ^19^F, ^23^Na, ^31^P, and ^133^Cs NMR techniques are discussed. The main attention is paid to the transport channel morphology, ionic hydration, charge group and mobile ion interaction, and translation ions and solvent mobilities in different spatial scales. Self-diffusion coefficients of protons and Li^+^, Na^+^, and Cs^+^ cations are compared with the ionic conductivity data. The microscopic ionic transfer mechanism is discussed.

## 1. Introduction

Ion exchangers, solid-state proton conductors, and polymer electrolytes for lithium batteries are very attractive in view of modern energy saving and development of environmentally friendly electrochemical technologies. Cation and anion polymeric ion-exchange membranes are widely applied in ion and liquid mixture separation (electrodialysis, brine electrolysis, extraction of rare metal ions from solutions, water–organic mixture separation, etc.) [1,2,3,4,5,6,7,8,9,10,11,12,13,14,15,16,17,18,19]. Since the beginning of the 21st century, interest in solid electrolytes has increased considerably due to the enhancement of fuel cells and high-power lithium batteries.

Progress in new efficient materials and technologies requires knowledge of ion and molecular transport mechanisms. The main results are concerned with macroscopic electro and mass transfer [1,2,3,4,5,6,7,8,9,10,11,12,13,14,15,16,17,18,19,20,21,22,23], whereas an investigation on a microscopic, molecular level is necessary. The relationship of the following factors should be revealed [24,25].

1. Construction of channels for mobile ions and molecules in the single nanometer range, because units of this dimension form the paths for macroscopic transfer.
2. An interaction of diffusing a substance with charge groups, known as ionic solvation.
3. A local diffusant mobility or diffusion jumps from one charge group to another and self-diffusion coefficients of ions and molecules.

NMR provides the possibility to obtain this information directly. The papers published thus far are devoted to ion and molecular transport in polymer ion-exchange membranes [24,25,26,27,28,29,30,31,32,33,34,35,36,37,38,39,40,41,42,43,44,45,46,47,48,49,50,51,52,53,54,55,56,57,58,59,60,61,62,63,64,65,66,67,68,69,70,71,72,73,74,75,76,77,78] and in inorganic solid ionic conductors [79,80,81,82,83,84,85,86,87,88,89]. High-resolution multinuclear NMR spectroscopy enables the study of ion surroundings and cation–anion interactions [24,25]. The chemical shift temperature and humidity dependence analysis of ^1^H water molecules provide an opportunity to calculate cation hydration numbers [24,25,28,29,30,31,32,34,35,36,37,41,60]. Multinuclear NMR spin relaxation makes it possible to measure ion and molecule local mobility frequencies [24,25,41,42,43,44,70,75,76,79]. Pulsed field gradient NMR (PFG NMR) is a unique technique of direct measurements of partial self-diffusion coefficients and relative diffusant amounts in an inhomogeneous medium. PFG NMR of ^1^H, ^2^H, ^7^Li, ^19^F, ^23^Na, ^31^P, and ^133^Cs is employed for a self-diffusion process characterized by solid electrolytes [24,25,26,27,32,33,34,35,36,37,38,40,41,42,44,45,46,47,48,49,51,52,53,54,55,56,57,58,59,60,73,77,78,79].

NMR diffusion experiments are very valuable because there is an opportunity to relate the ion translation mobility to the ionic conductivity, which is a very important charge-transfer macroscopic parameter. Details of NMR technique applications to solid electrolytes are given in many papers, which may guide persons who are going to apply NMR [24,25,41].

This review is aimed at showing NMR abilities for ionic and molecular transport characterization in solid electrolytes. First of all, attention is paid to polymer perfluorinated sulfo cation Nafion membranes and membranes based on sulfonated polystyrene. Anion exchangers and membranes are also considered. Water, alcohol, and alkaline ion diffusion is discussed. Self-diffusion data are compared with ion conductivities. The perfluorinated sulfo cation membrane Nafion is selected as a model system, the investigation of which gives a fundamental concept of ionic and water transport in different spatial scales. A special anhydrous mechanism of proton transport in inorganic composite materials based on cesium acid sulfates and phosphates is described. Recently, the trend of synthesis and research, including NMR methods, of the composites with other inorganic compounds, such as dopants, to increase ionic conductivity has been widely developed [80,81]. High-resolution NMR, NMR relaxation, and PFG NMR provide the possibility to reveal the mechanism of proton conductivity in the composite based on 12-phosphotungstic acid and its acid salts [21,22,23,79].

We do not discuss NMR applications to polymer electrolytes for lithium batteries since it is out of scope of this review. Some recent papers devoted to NMR applications of these electrolytes are listed in references [90,91,92,93,94,95,96,97,98,99,100,101].

## 2. Membrane Nanostructure

The first step to reveal the ionic transfer mechanism is a nanostructure of ionic transport channels. Nafion membranes attract keen interest.

**Figure 1:**
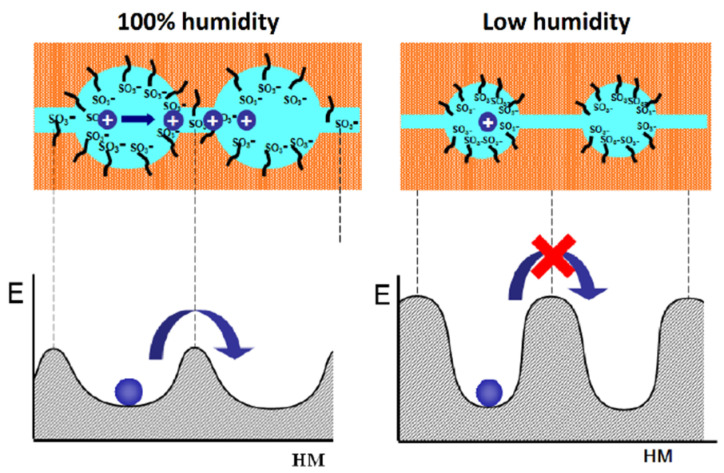
Schematic representation of the cluster Gierke model. Left is maximum humidity. Right is low humidity.

The so-called Gierke model was proposed on the basis of small angle X-ray scattering (SAXS) data. According to this model, sulfonate groups, mobile cations, and water molecules are associated in spherical clusters with a diameter of 5 nm or less depending on membrane humidity. The clusters connect to each other by narrow channels formed by solitary sulfonate groups, and the channel width is about 1 nm (Figure 1) [102].

**Figure 2:**
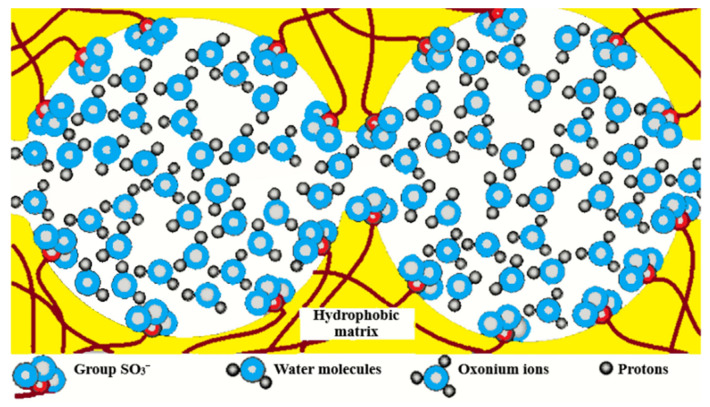
Schematic representation of Gierke model. Reprinted with permission from Ref. [7]. Copyright © 2022 Pleiades Publishing, Ltd.

**Figure 3:**
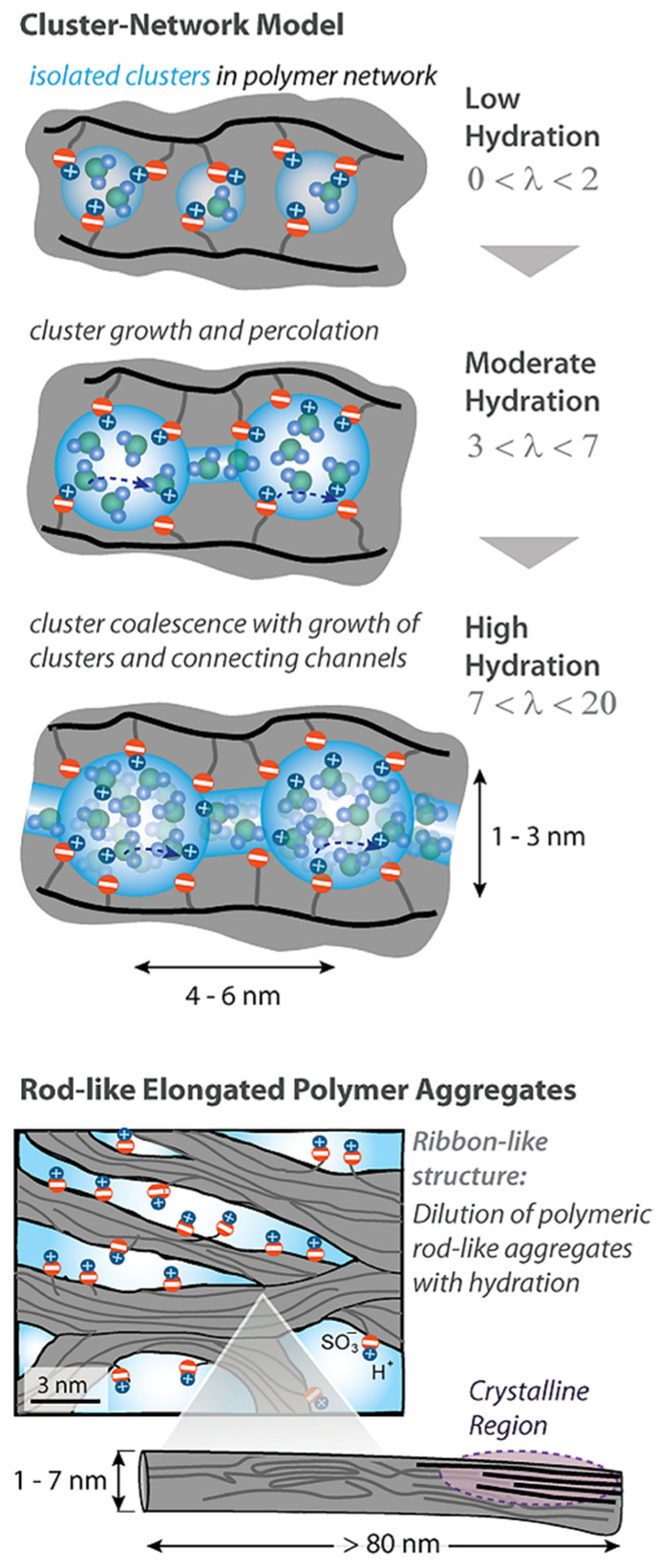
Proposed morphological descriptions for Nafion (or PFSA) [58]: Cluster-network model (by Gierke, Hsu, and co-workers), evolution of morphology from spherical domains to rod-like aggregates in dispersion (by Gebel). Copyright © 2022 American Chemical Society.

The cluster diameter is the only experimentally measured characteristic, but no channels were observed. Some modifications of the Gierke model are shown in Figure 2 and Figure 3.

As indicated in Figure 3, at low hydration where *λ* < 2 (*λ* is the amount of water molecules per sulfonate group) the macroscopic transport is blocked because the channels “dry up”. At higher humidity, the cluster diameter increases and channels are opened. The macroscopic water and cation transfer is limited by their passing in narrow channels. The Gierke model describes qualitatively the threshold effect of the diffusion coefficient and conductivity humidity dependences. The channel width is about 1 nm, but the distance between sulfonate groups in the channel is more comparable with the average distance (0.7 nm);, therefore, the hydrogen bonds between water molecules in the channel are destroyed, which is the cause of low water and cation mobility, whereas the molecular and ionic mobility in hydrated clusters should be much higher. The first measurements of water self-diffusion coefficients by pulsed field gradient NMR show that the macroscopic self-diffusion coefficient in a Nafion membrane is comparable with the coefficient of bulk water. The correlation times of local water molecules and Li^+^ cation translation motions in the clusters were estimated based on the ^1^H and ^7^Li NMR spin relaxation. The self-diffusion coefficients obtained by PFG NMR are macroscopic, because during the measurement time the particles move at least one hundred nm, passing through the clusters and channels many times. Their values were comparable with the self-diffusion coefficients calculated using the Einstein Equation (1).

(1) $$D=\frac{l^{2}}{6\cdot \tau_{i}}$$

where *l* is the jump length of a water molecule (0.3 nm) or Li^+^ cation (0.7 nm, average distance between SO_3_^−^ groups), *D* is the diffusion coefficient, and *τ_i_* is the jump time.

As it is shown in Table 1, the macroscopic experimental *D*^exp^ and microscopic calculated *D*^calc^ self-diffusion coefficients of water molecules and lithium cations are in good agreement, which is not confirmed by the Gierke model.

**Figure 4:**
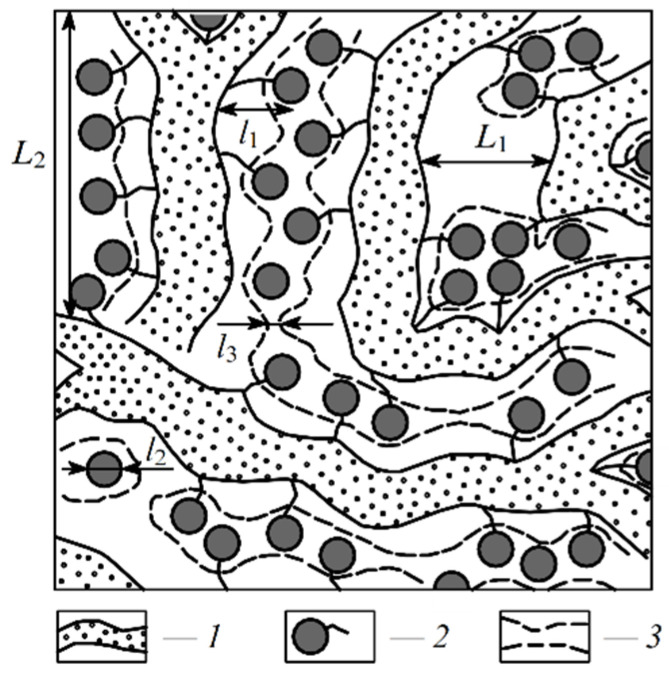
Structure of the amorphous part of the perfluorinated sulfonate cation-exchange membrane. (1) Polymer backbone; (2) hydrated counterions and functional groups at a low moisture content; (3) transport channels for ions and water molecules at a high moisture content; *L*_1_ = 4 nm according to small angle X-ray scattering (SAXS) data; *L*_2_ = 10 nm according to Mössbauer spectroscopy; *l*_1_ = l.2–1 nm according to ENDOR and relaxation NMR data; *l*_3_ = 1.5 nm according to standard porosimetry and ENDOR methods (adapted from [103]).

Another model was developed on the basis of detailed SAXS measurements accompanied by Mössbauer spectroscopy, ENDOR, NMR relaxation, and standard porosimetry data (Figure 4).

**Figure 5:**
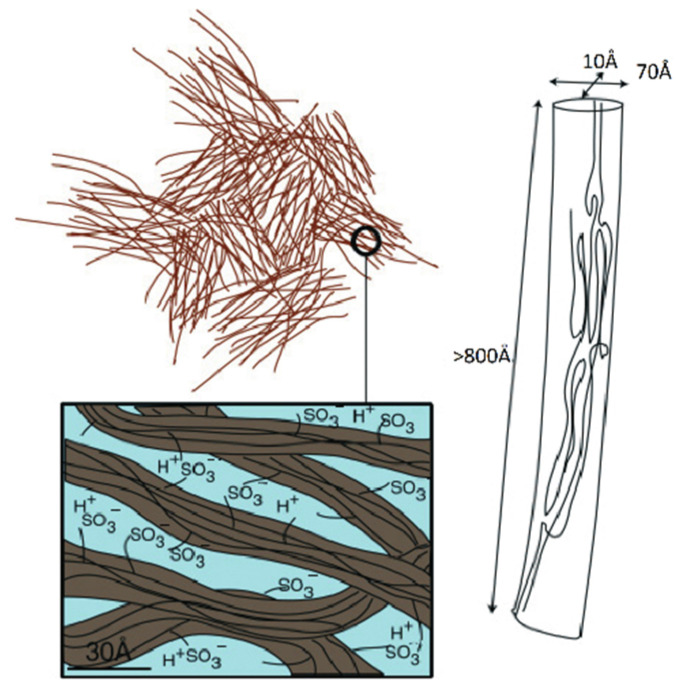
New insight of a Nafion membrane multiscale, structural model for Nafion membranes. Rod-like polymer aggregations (adapted from [58,104]).

According to this nanostructure model, the macroscopic molecular and ionic transfer is controlled by water molecule and cation local jumping, which is in good agreement with the results in Table 1. This model was confirmed by the authors of [104] who proposed rod-like aggregates (Figure 5).

**Figure 6:**
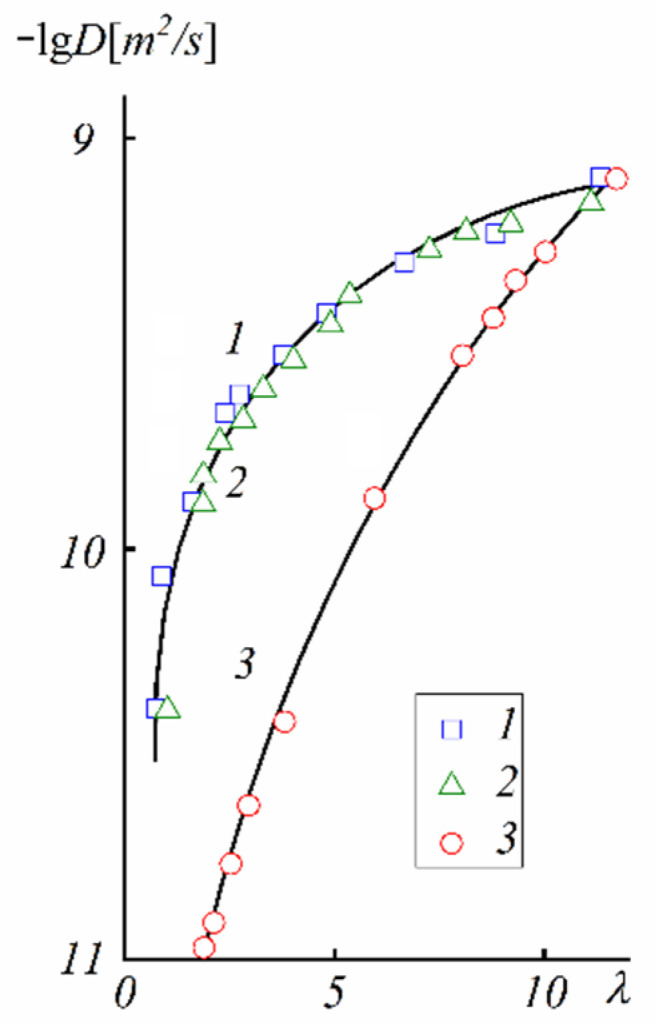
Humidity dependences of the water self-diffusion coefficients in the acid ionic form of the sulfo cation exchanger CU-2-8 (Russian analog of Dowex 50 W). 1, 2, calculated from Equation (1); 3, measured by PFG NMR. 1, sample swelled in H_2_O; 2, sample swelled in D_2_O, signal of residual protons was recorded (adapted from [33]).

The next type of membrane is a membrane with sulfonate groups binding with styrene fragments. For these types of compositions, the calculated self-diffusion coefficient is higher compared with the macroscopic diffusion coefficient, as it is shown in Figure 6, where the dependences of water self-diffusion coefficients on humidity *λ* are shown for the sulfonated exchanger CU-2-8 (Russian analog of Dowex 50 W). Therefore, the cluster channel model of the nanostructure (particularly, the model in Figure 2) is suitable for ionic molecular transfer explanation. This model is especially reasonable for ion exchangers based on styrene–divinylbenzene crosslinked copolymers. Namely, rather narrow channels are formed in the crosslinked fragments of polystyrene chains [33].

As mentioned above, it should be concluded that Nafion macroscopic transport is carried out by local jumping of water molecules and cations between neighboring sulfonate groups. Therefore, ion transport is determined by ionogenic group hydration. For this reason, the hydration character should be correlated with the translational diffusion mobility of water molecules and cations, as well as membrane ionic conductivity.

## 3. Ion Hydration

The characteristic property for ion-exchange membranes is the threshold dependence of water and counterion mobility on the humidity [24,25,33,35,36]. For example, in a Nafion membrane, the water and cation translational mobility as well as the ionic conductivity decrease by two or three orders of magnitude if the water content *λ* decreases from 10–15 to 3–5 molecules per sulfonate group [24]. Therefore, hydration controls the ion mobility in membrane nanochannels. The water content of the membranes is determined by the cation hydration energy and, therefore, water molecules are mainly sorbed by cations [24,25]. The basic cation hydration characteristic is the hydration number *h*, which is the amount of water molecules in the first coordination sphere of the cation. For water content where *λ* > *h*, the water molecule located between the sulfonate group and hydrated cation–anion ionic pair dissociates and, therefore, the cation is mobile and its surroundings are similar to an aqueous solution. If *λ* < *h*, the oxygen atom of the sulfonate group is involved in the cation of the nearest sphere and the cation is fixed on the charged group. This phenomenon explains sharp ion mobility–humidity dependence.

**Figure 7:**
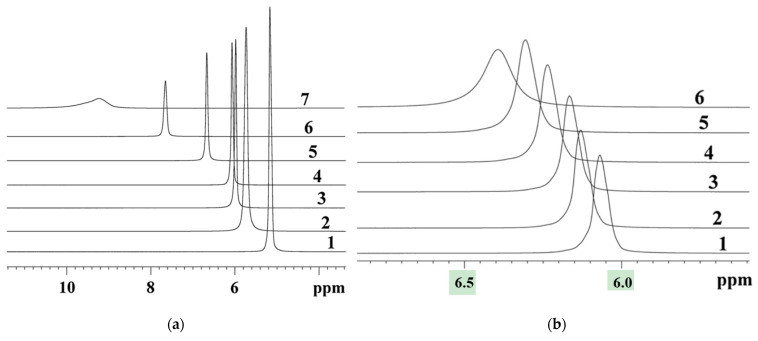
(**a**) The ^1^H NMR spectra of the acid ionic form of the Nafion 117 membrane at different humidities: (1) *RH* = 95%; (2) *RH* = 78%; (3) *RH* = 64%; (4) *RH* = 58%; (5) *RH* = 32%; (6) *RH* = 10%; (7) *RH* = 0. (**b**) The ^1^H NMR spectra of the acid ionic form of Nafion membranes at different temperatures: (1) +25 °C; (2) +10 °C; (3) 0 °C; (4) −20 °C; (5) −40 °C; (6) −60 °C. Membrane samples were equilibrated with water vapor at 58% relative humidity. Reprinted with permission from Ref. [41]. Copyright © 2022 Springer-Verlag GmbH Austria, part of Springer Nature.

High-resolution ^1^H NMR of water molecules is a direct technique for the calculation of the hydration number. Depending on the cation type (H^+^ or metal cation), the water chemical shift in membranes varies from 6 ppm to −2 ppm (relative to bulk water). For a standard NMR spectrometer with a 500 MHz ^1^H NMR frequency, this variation is from 3000 to −1000 Hz. As it is shown in Figure 7, the ^1^H NMR lines are narrow even at low temperature and humidity, thus chemical shift may be measured with high accuracy.

The hydration number is calculated from the ^1^H chemical shift dependence on temperature. The physical meaning of this calculation is as follows. Ion hydration water molecules are fixed, but their hydrogen bonds are destroyed; hence, the chemical shift is temperature independent. Therefore, only water molecules of the next hydration spheres, whose hydrogen bond network is similar to the bulk water network, are temperature dependent. The fast molecular exchange occurs between water molecules in different positions. Therefore, the average singlet line is observed with the following chemical shift:

(2) $$\delta =p_{h}\cdot \delta_{h}+p_{H_{2}O}\cdot \delta_{H_{2}O}\;$$

where *δ_h_* is the chemical shift of protons of the hydrated cation, *p_h_* is the relative part of hydrated cation protons; $\delta_{H_{2}O}$ is the bulk water chemical shift, and $p_{H_{2}O}$ is the bulk water relative part. The hydration numbers can be calculated from Equations (3) and (4) [25,41]. For the acid membrane ionic form:

(3) $$h=\lambda -\frac{\left(0.5+\lambda \right)\cdot \frac{d\delta }{dT}}{\frac{d\delta_{H2O}}{dT}}$$

For the salt membrane ionic form:

(4) $$h=\lambda \left[1-\frac{\frac{d\delta }{dT}}{\frac{d\delta_{H2O}}{dT}}\right]\;$$

where *dδ*/*dT* is the temperature dependence of the membrane water ^1^H chemical shift and *dδ_H_*_2*O*_/*dT* is the bulk water ^1^H chemical shift temperature dependence.

The calculation procedure of the hydration number from the ^1^H chemical shift temperature dependence was described in detail in [24,25,30,32,33,35,41].

**Figure 8:**
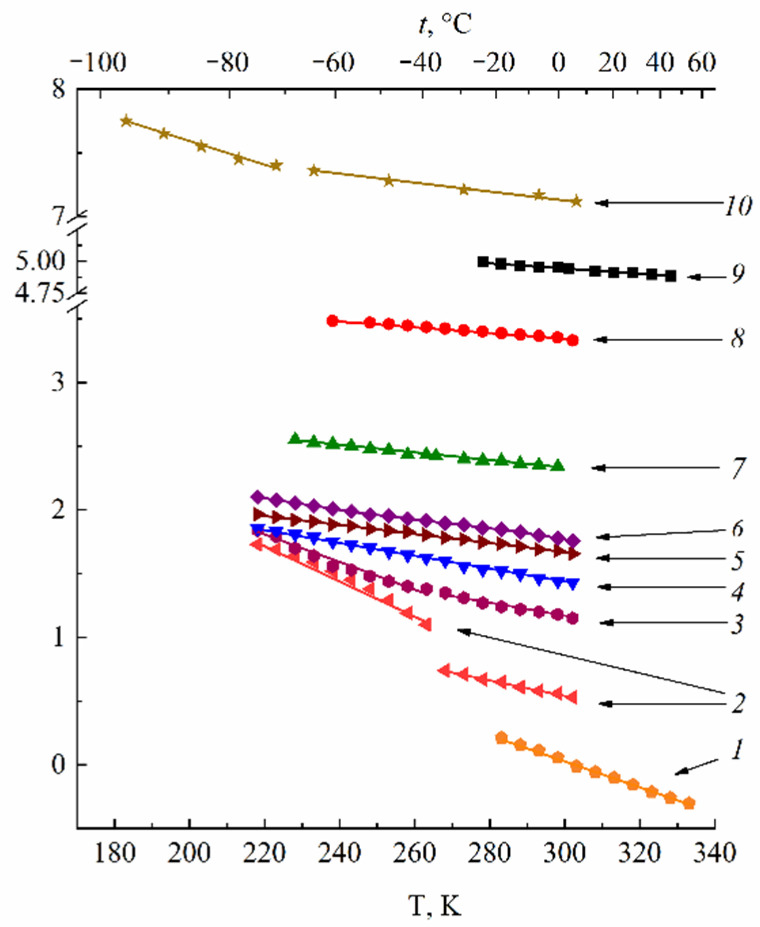
Temperature dependences of ^1^H NMR chemical shifts in acid ionic form of the Nafion membrane at different water contents (*δ*_H2O_ is the bulk water chemical shift equal to 4.30 ppm relative to TMS, at 20 °C). (1) Bulk H_2_O; (2) *RH* = 98% (*λ* = 17.5 ± 0.4); (3) *RH* = 95% (*λ* = 12 ± 0.4); (4) *RH* = 78% (*λ* = 7.4 ± 0.4); (5) *RH* = 64% (*λ* = 6.4 ± 0.4); (6) *RH* = 58% (*λ* = 5.8 ± 0.4); (7) *RH* = 32% (*λ* = 4.4 ± 0.4); (8) *RH* = 10% (*λ* = 3.2 ± 0.4); (9) *RH* = 0% (*λ* = 1.9 ± 0.4, drying on P_2_O_5_ or at 110 °C); (10) is the chemical shift temperature dependence in acid ionic form of the Nafion NRE-212 membrane. Adapted from [41,75].

The proton chemical shift temperature dependences in the Nafion acid form are shown in Figure 8.

These dependences are straight lines from −55 °C to 50 °C for *λ* < 7.4 (curves 4–9). For higher *λ*, this dependence slope increases in the freezing temperature range (curves 2 and3, Figure 8). The same shape of the chemical shift temperature dependence was observed for the Nafion NRE-212 membrane (curve 10, Figure 8) [75].

The authors of [75,76] explain the shift of the ^1^H peak to a lower magnetic field with decreasing temperature by the enhancement of intermolecular interactions via hydrogen bonds.

**Figure 9:**
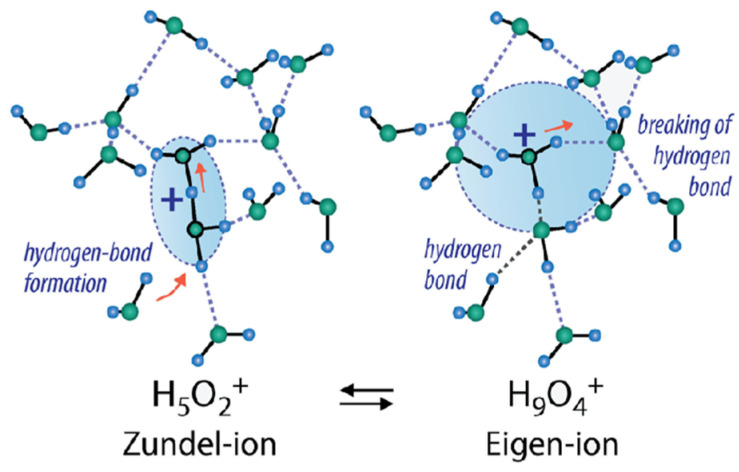
Hydrated proton structure at low humidity (left) and high humidity (right). Reprinted with permission from Ref. [58]. Copyright © 2022 American Chemical Society.

In the acidic ionic form, the ^1^H line width is narrow even after the Nafion membrane was dried to a constant weight, indicating a high proton mobility under these conditions. The direct NMR measurements of the water content show that two residual water molecules remained after drying to form the hydroxonium (Zundel) ion H_5_O_2_^+^, which explains a high proton mobility [41]. In Table 2, the H^+^ hydration numbers calculated from Equation (3) are given. The absolute value *λ* with a glance at two residual water molecules is indicated in Figure 8 and Table 2. At a low water content (*λ* < 3–4), *h* is about 2, but with increasing humidity (*λ* > 6) H_9_O_4_^+^ (Eigen-ion) is formed (Figure 9) [58].

It should be mentioned that this way of calculating the hydration number is rather crude, but some principal conclusions can be drawn, as it is shown above. These results are fundamental, because the same hydration peculiarities have been observed for all sulfonic cation exchangers [24,31,32,41].

**Figure 10:**
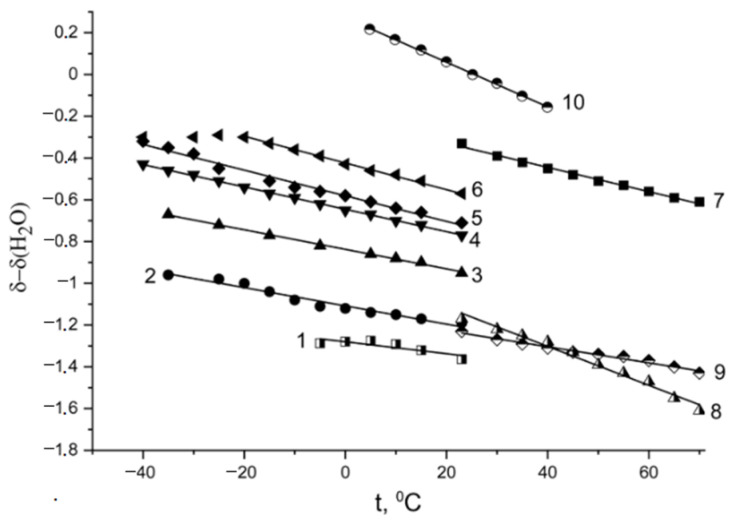
Temperature dependences of the water proton chemical shifts in the Li^+^, Na^+^, and Cs^+^ ionic forms of Nafion membranes at various relative humidities, where *δ*_H2O_ is the bulk water chemical shift *δ*_H2O_ = 4.30 ppm relative to TMS, at 20 °C; Li^+^ ionic form: (1) *λ* = 0.9, (2) *λ* = 2.0, (3) *λ* = 4.0, (4) *λ* = 5.7, (5) *λ* = 7.4, (6) *λ* = 10.7, (7) *λ* = 12; (8) Na^+^ ionic form: *λ* = 10; (9) Cs^+^ ionic form: *λ* = 4; (10) bulk water; *λ* is the amount of water molecules per sulfonated group. Reprinted with permission from Ref. [60]. Copyright © 2022 Elsevier B.V. All rights reserved.

The hydration numbers of alkaline and alkaline-earth metal ions were also calculated. In Figure 10, the ^1^H chemical shift temperature dependences for Li^+^, Na^+^, and Cs^+^ Nafion ionic forms are shown.

In Table 3, the *h* values of Li^+^ calculated from Equation (4) at different *λ* are indicated. As shown in Table 3, at *λ* < 10.7 hydration number *h* of the Li^+^ cation is lower than (4), which is equal to *h* for aqueous salt solutions. Hydration numbers for Na^+^ and Cs^+^ cations at the maximum water content are 6 ± 1 (*λ* = 10 at 98% *RH*) and 1 ± 0.2 (*λ* = 4 at 98% *RH*), respectively [60].

Another way of estimating the cation hydration number is an analysis of the water ^1^H chemical shift humidity dependences [25,31,32,33,35,36,41].

**Figure 11:**
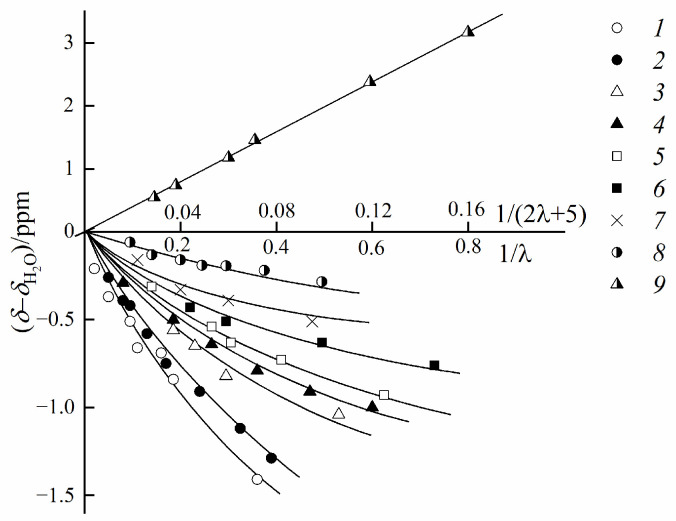
Dependences of chemical shifts of water on the moisture content of the MF-4SC membrane. Membrane ionic form: (1) Li^+^, (2) Na^+^, (3) K^+^, (4) Rb^+^, (5) Cs^+^, (6) Ba^2+^, (7) Ca^2+^, (8) Mg^2+^, (9) H^+^ (adapted from [36]).

In Figure 11, these dependences for H^+^, alkaline, and alkaline-earth metal cations in the perfluorinated sulfo cation-exchange membrane MF-4SC are shown.

Hydrogen numbers, hydration heat, and a part of the cleaved hydrogen bonds, as a result of cation polarization, are given in Table 4. The *h* value, as well as a part of the cleaved hydrogen bonds, increases with increasing hydration energy.

**Figure 12:**
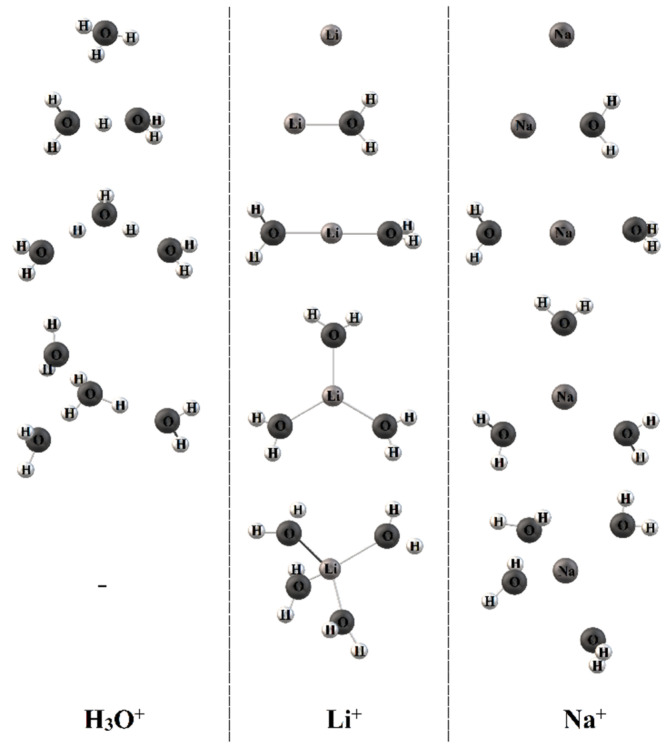
Optimized geometries of the X^+^(H_2_O)*_n_* clusters, where X^+^ is H^+^, Li^+^, and Na^+^ cations. Reprinted with permission from Ref. [74]. Copyright © 2022 American Chemical Society.

Some examples of the cation hydrated structure in Nafion 117, Flemion SH-120 (EW 909), and LSH-180 (EW 1099) are shown in Figure 12.

High-resolution ^7^Li, ^23^Na, and ^133^Cs NMR provides the possibility to calculate a part of the cations Li^+^, Na^+^, and Cs^+^ interacting with SO_3_^−^ membrane groups and directly forming contact ionic pairs. The NMR spectrum of these cations is a singlet line, whose chemical shift is the average value of chemical shifts of a cation in contact and separate ionic pairs.

(5) $$\delta =p_{c}\cdot \delta_{c}+p_{s}\cdot \delta_{s}\;$$

where *δ_c_* is the chemical shift of a cation in the contact pair, *p_c_* is a relative part of the contact ionic pairs, *δ_s_* is the chemical shift in the separate ionic pair, and *p_s_* is a part of the separate ionic pairs. Additionally, *δ_c_* is the chemical shift at low humidity when all cations are fixed to SO_3_^−^ groups and *δ_s_* is the chemical shift of a fully hydrated cation. Cation NMR linewidth is the widest for contact ionic pairs and narrowest for separate ionic pairs. The linewidth is inverse to the cation mobility 1/*τ_d_*, where *τ_d_* is the quadrupole interaction correlation time.

**Figure 13:**
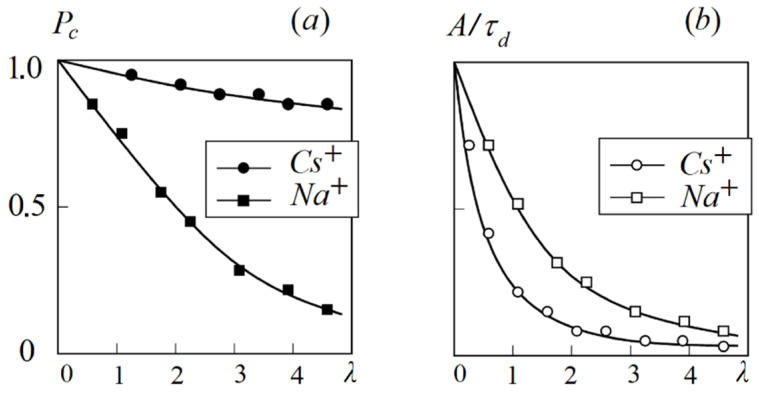
(**a**) Relative part of contact ionic pairs *P_c_* of Na^+^SO_3_^−^ and Cs^+^SO_3_^−^; (**b**) relative translational mobility frequency *A*/*τ_d_* of Na^+^ and Cs^+^ cation humidity dependences in the perfluorinated sulfo cation-exchange membrane MF-4SC [105].

In Figure 13, the dependences of contact ionic pairs *P_c_* (a) and relative ionic mobility *τ_d_* (b) of Na^+^ and Cs^+^ cations on the water molecule amount per SO_3_^−^ group *λ* is shown. The value of *P_c_* is lower, but the translation jumping frequency *A*/*τ_d_* is higher for the sodium cation as compared to the cesium cation.

**Figure 14:**
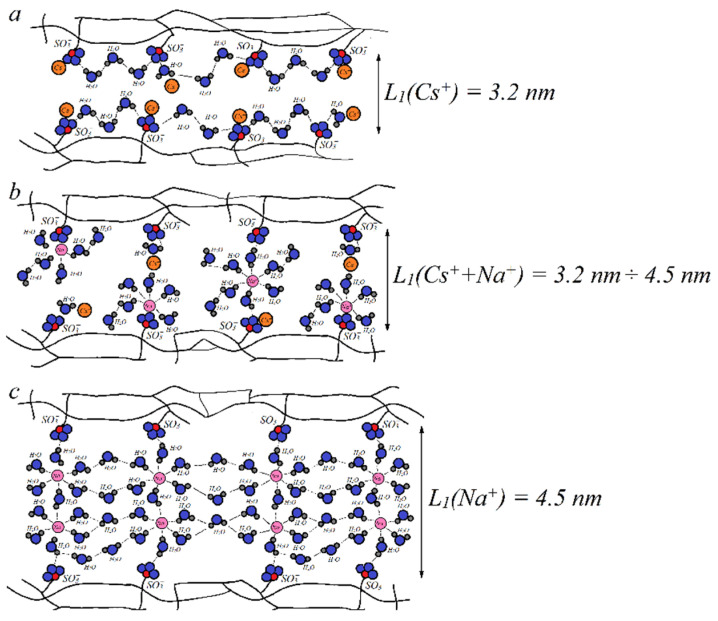
Schematic representation of the Nafion membrane ionogenic channel fragment. Membrane ionic form: (**a**) Cs^+^, (**b**) mixture Cs^+^/Na^+^, (**c**) Na^+^, where 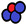 is a SO_3_^−^ group, 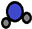 is a water molecule, 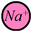 is a Na^+^ cation, 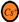 is a Cs^+^ cation, and *L*_1_ is the channel width [105].

Let us examine the mixture of the Nafion ionic form containing high hydration energy Na^+^ cation and low hydration energy Cs^+^ cation. In the mixed ionic form, the average water content at the maximum humidity is lower than that for the individual Na^+^ form, but higher than that for the individual Cs^+^ form. Therefore, by the variation of Na^+^ and Cs^+^ concentrations we can change the average value of *λ.* Thus, as shown in Figure 13, it is possible to vary the relative part of contact or separate ionic pairs for each cation. For example, we can make conditions for Na^+^ to form contact ionic pairs, but Cs^+^ and SO_3_^−^ are separated by the water molecule (Figure 14b). Therefore, the cesium cation mobility is higher compared to the sodium cation mobility. The channel width *L_1_* increases with increasing water content *λ.* The value of *λ* in the mixed Na^+^ + Cs^+^ ionic form is lower than that in the Na^+^ form, but higher than that in the Cs^+^ form. Therefore, the *L*_1_ value in the mixed form is between the channel width of individual Na^+^ and Cs^+^ forms. Thus, another reason for increasing Cs^+^ mobility in the Na^+^ + Cs^+^ ionic form is a higher width diffusion channel compared with individual Cs^+^ (Figure 14a,b). The narrowing of the channel width *L*_1_ in the mixed ionic form opposite to the individual Na^+^ form is an additional reason for decreasing sodium cation mobility (Figure 14b,c). Therefore, the detailed NMR investigations gave an opportunity to understand the microscopic mechanism of membrane selectivity of sulfo-containing membranes in regard to the alkaline metal cations. These results may be a guide for the development of new materials.

The water and cation behavior at subfreezing temperatures is controlled by membrane hydration particularities. The DSC shows a peak at temperatures below 0 °C, which is explained by freezing of the so-called “unbounded” water forming the ice phase, whereas another part of water (“bounded” water) remains mobile [41,106]. The amount of these two types of water is calculated from the DSC results [106].

**Figure 15:**
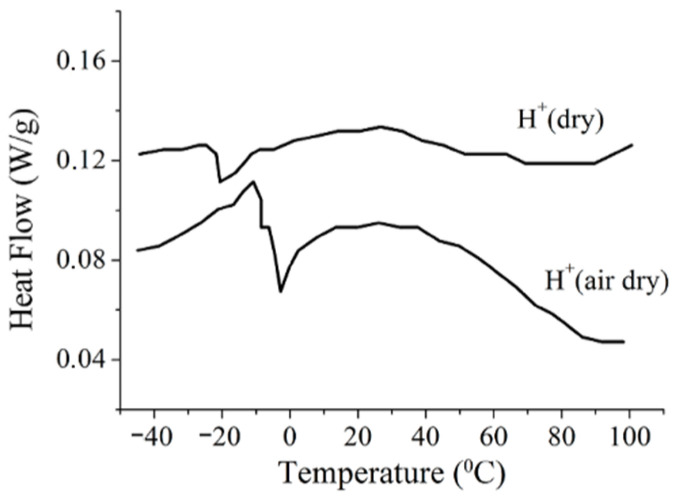
DSC thermograms in H^+^ ionic forms of perfluorinated sulfo cation-exchange MF-4SC membrane, which is a complete analog of Nafion. “Dry” means that the sample was dried to a constant weight at 120 °C (*λ* is about 2 water molecules per sulfonate group), “air dry” means the sample dried to a constant weight at room temperature (*λ* is about 5 water molecules per sulfonate group). Adapted from [44].

**Figure 16:**
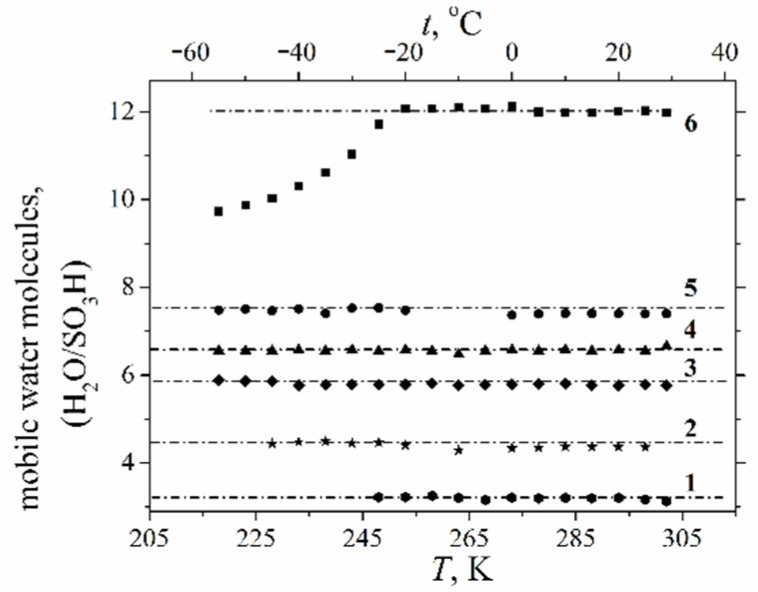
Dependences of the mobile water molecule amount in the acidic form of the Nafion 117 membrane on temperature at different water contents: (1) *λ* = 3.2 ± 0.4; (2) *λ* = 4.4 ± 0.4; (3) *λ* = 5.8 ± 0.4; (4) *λ* = 6.4 ± 0.4; (5) *λ* = 7.4 ± 0.4; (6) *λ* = 12 ± 0.4. Reprinted with permission from Ref. [41]. Copyright © 2022 Springer-Verlag GmbH Austria, part of Springer Nature.

High-resolution ^1^H NMR of water molecules is a direct way to calculate the number of mobile water molecules, whereas no NMR spectrum of ice water is observed. It was shown that starting from *λ* ≈ *h* a mobile water amount does not decrease at a low temperature [56,75,76]. This result agrees with the DSC data: with decreasing humidity only bounded water remains, which cannot form ice because its molecules form hydrogen bonds with the polymeric matrix; therefore, the DSC peak should disappear. As shown in Figure 15, the DSC signal is observed even in a dry sample at *λ* = 2, whereas the molecules remain mobile until −60 °C at *λ* ≤ 10 (Figure 16).

**Figure 17:**
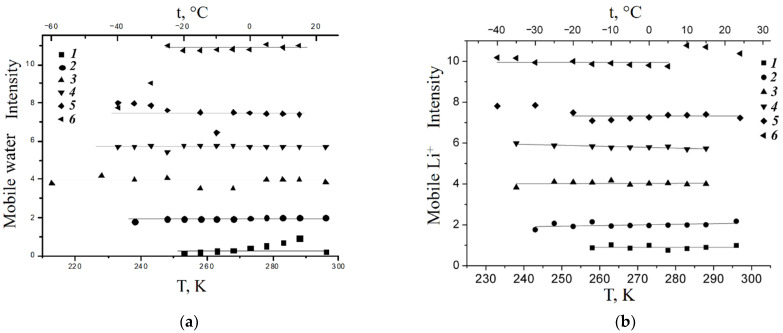
Dependences of the number of mobile water molecules per sulfonated group *λ* (**a**) and mobile Li^+^ cations (**b**) on temperature in the lithium ionic form of the Nafion 117 membrane; (**a**) amount of mobile water molecules per sulfonate group: (1) *λ* = 0.9, (2) *λ* = 2.0, (3) *λ* = 4.0, (4) *λ* = 5.7, (5) *λ* = 7.4, (6) *λ* = 10.7. (**b**) Relative intensity of ^7^Li^+^ mobile signals: (1) *λ* = 0.9, (2) *λ* = 2.0, (3) *λ* = 4.0, (4) *λ* = 5.7, (5) *λ* = 7.4, (6) *λ* = 10.7. Reprinted with permission from Ref. [24]. Copyright 2021 MDPI.

At higher *λ*, water is adsorbed in macropores and forms the ice phase at the freezing temperature [41,107]. The similar phenomenon was observed in the Li ionic form of the Nafion membrane (Figure 17) [24,60] and in the membrane based on polystyrene [34].

As shown in Figure 17, water and Li^+^ cations are mobile at −40 °C at *λ* ≤ 10.7.

The discrepancy between the DSC and NMR data was explained on the basis of the ^1^H spin-relaxation data [25,44]. It was concluded that at the freezing temperature, water molecules form additional hydrogen bonds with the formation of associates, which remain mobile. The formation of these hydrogen bonds is accompanied by the appearance of the DSC peak. This explanation is confirmed by the ^1^H chemical shift temperature dependences. As shown in Figure 7 and Figure 8, the slope of the chemical shift temperature dependence curves increased below 0 °C, which indicates the formation of extra hydrogen bonds.

**Figure 18:**
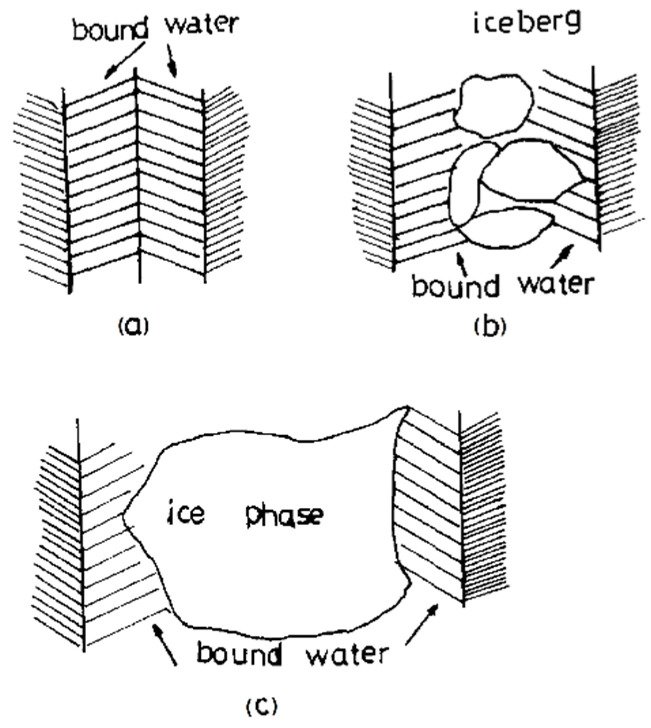
Schematic fragments in two channelsC at the temperature below 0 °C: (**a**) narrow pores (2–10 nm)—water is bound to the pore walls and cannot transform into ice; (**b**) increased pore width—water molecules, that are far from the pore walls, tend to transform into microcrystals of ice; (**c**) a separate ice phase. Reprinted with permission from Ref. [108]. Copyright © 2022 Published by Elsevier B.V.

The NMR method was applied to a water freezing investigation in pores of cellulose acetate membranes. As shown in Figure 18, in narrow pores (2–10 nm) water is bounded with pore walls and cannot transform into ice (Figure 18a). With increasing pore width, water molecules located far from pore walls tend to transform into microcrystals of ice (Figure 18b) and a separate ice phase (Figure 18c) [107].

## 4. Water and Cation Self-Diffusion

The water and cation self-diffusion coefficients were measured by pulsed field gradient technique (PFG NMR). The stimulated echo sequence was used.

The evolution of spin echo signal (diffusion decay) is described by the following equation:

(6) $$A\left(g\right)=A\left(0\right)\exp\left(-\gamma^{2}g^{2}\delta^{2}t_{d}D_{s}\right),\;$$

where *γ* is the gyromagnetic ratio of ^1^H, ^7^Li, ^19^F, ^23^Na, and ^133^Cs; *g* is the gradient pulse amplitude; *δ* is the gradient pulse duration; *t_d_* = Δ−*δ*/3 is the diffusion time; Δ is an interval between gradient pulses; and *D* is the self-diffusion coefficient. The details of self-diffusion coefficient PFG NMR measurements are given in [24,25,37,41,46,100,108].

**Figure 19:**
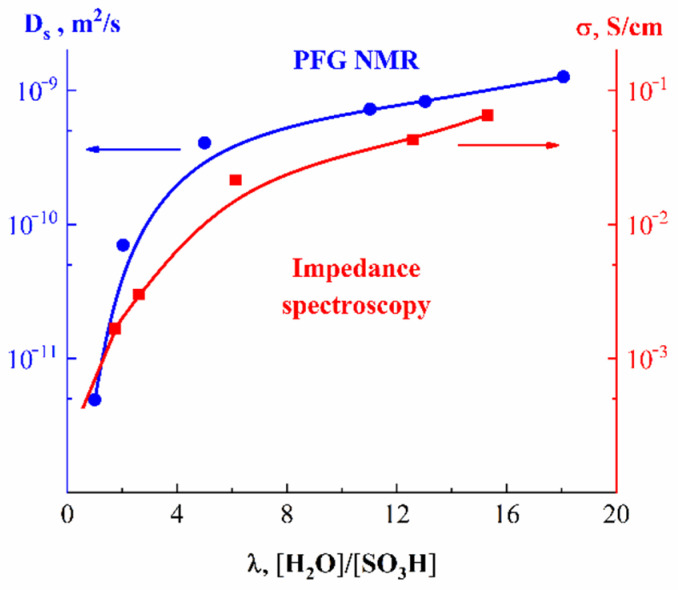
Dependences of the water self-diffusion coefficient and proton conductivity on humidity (*λ*), where *λ* is the amount of water molecules per sulfonate group in the acid form of the MF-4SC membrane. Reprinted with permission from Ref. [24]. Copyright 2021 MDPI.

**Figure 20:**
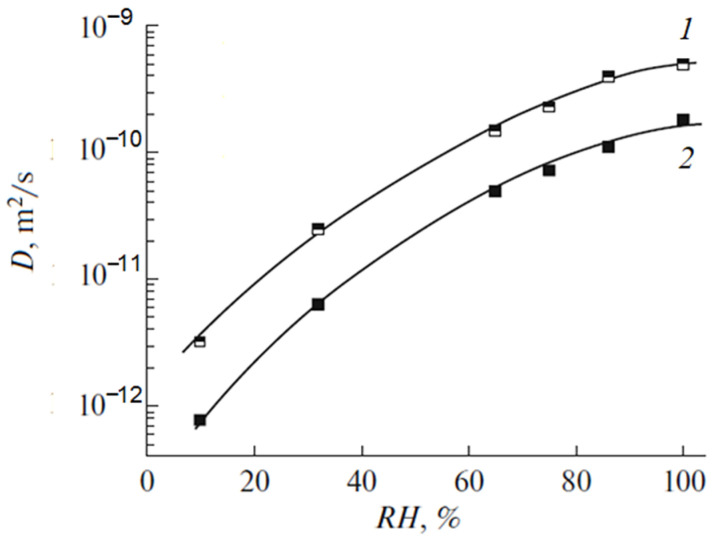
Average diffusion coefficients of water molecules and H^+^ cations measured by pulsed field gradient NMR (1) and calculated from the proton conductivity data according to Equation (7) (2) in the H^+^ form of the MSC membrane at different moisture contents. When *RH* = 100% the membrane is in contact with water. Reprinted with permission from Ref. [34]. Copyright © 2022 Pleiades Publishing, Ltd.

In Figure 19, the humidity dependences of the water self-diffusion coefficients measured by pulsed field gradient NMR and proton conductivity in the perfluorinated sulfo cation-exchange MF-4SC membrane are shown. These dependences are similar, indicating that the ionic transfer is controlled by the water translational mobility. The same character was observed for other membranes. In Figure 20, the average water self-diffusion coefficients and diffusion coefficients calculated from the Nernst–Einstein dependences (Equation (7)) on the relative humidity are given for the membrane based on polyethylene and grafted sulfonated polystyrene (MSC) [109,110,111].

(7) $$\sigma ={ne}^{2}\frac{D}{kT},$$

where *n* is the number of charge carriers, cm^3^; *D* is the self-diffusion coefficient, m^2^/s; *e* is the electron charge, 1.9 × 10^–19^ C; *k* is the Boltzmann constant, 1.38 × 10^–23^ J/K; and *T* is the temperature.

**Figure 21:**
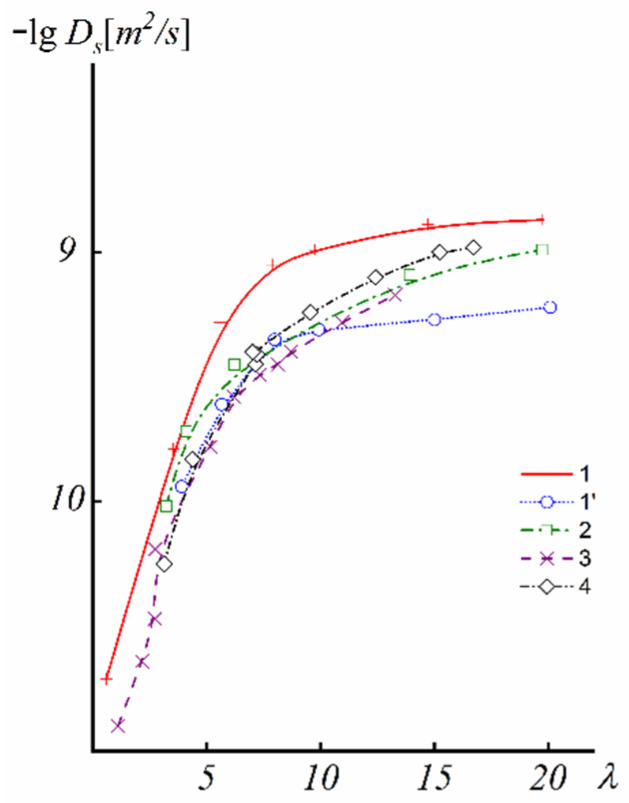
Water self-diffusion coefficient humidity dependences: (1,1′) H^+^ ionic form of the membrane MC-44; (2) Na^+^ ionic form of the MC-40 membrane; (3) H^+^ ionic form of the MC-40 membrane; (4) Na^+^ ionic form of the macropore sulfo cation exchanger CU-23. Adapted from [33].

These dependences are symbate. The ionic conductivity humidity dependence also shows the threshold character. The threshold shape dependences of the water self-diffusion coefficients on humidity are observed for all types of ion exchangers. For example, the water self-diffusion coefficient humidity dependences for a sulfo cation exchanger based on the copolymer of styrene and divinylbenzene MC-44, sulfonated polystyrene membrane MC-40, and macropore sulfo cation exchanger are shown in Figure 21. These dependences are very similar for all ion exchangers.

The water self-diffusion coefficients are essentially higher compared with the calculated diffusion coefficients. This difference will be discussed below.

**Figure 22:**
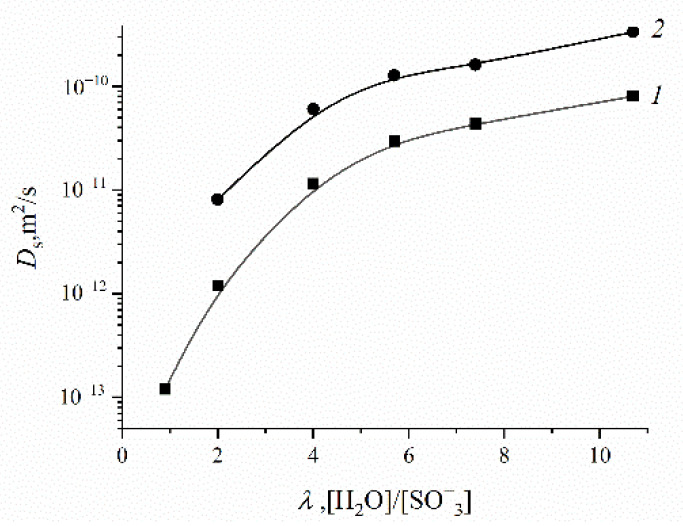
Self-diffusion coefficients of Li^+^ ion (curve 1) and water molecule (curve 2) dependences on the water content *λ* in the Nafion 117 membrane Li^+^ ionic form, where *λ* is the water amount per sulfonate group. Reprinted with permission from Ref. [60]. Copyright © 2022 Elsevier B.V. All rights reserved.

The direct measurement of the Li^+^ cation self-diffusion has shown that the cation and water molecule self-diffusion coefficient humidity dependences are also similar (curve 1 and curve 2 in Figure 22).

**Figure 23:**
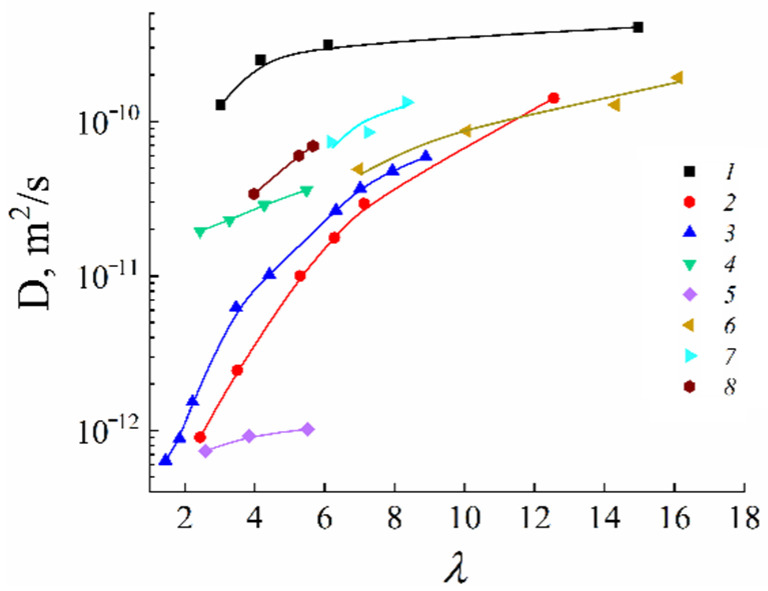
Diffusion coefficients of water molecule dependence on the moisture content for sulfonate cation-exchange resin MF-4SC (1–4) and carboxylic F-4CF (5–8) perfluorinated membranes in various ionic forms. Membrane ionic forms: (1, 5) H^+^, (2, 6) Li^+^, (3, 7) Na^+^, (4, 8) Cs^+^. Adapted from [24,38].

**Figure 24:**
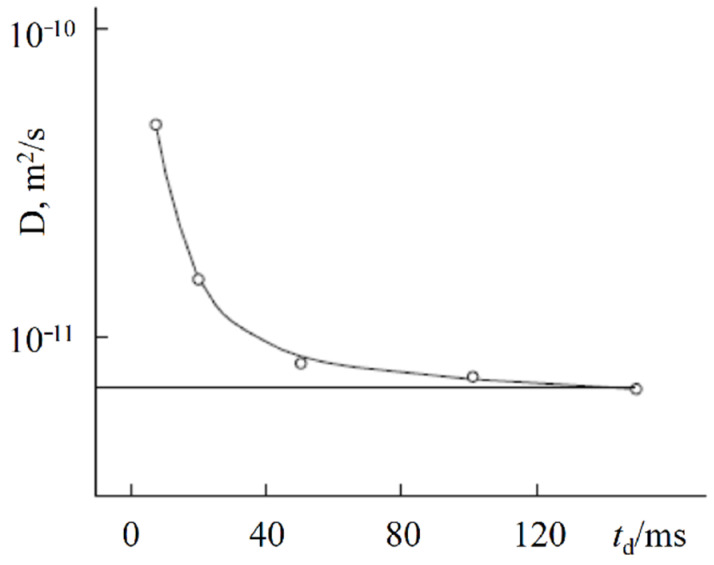
Average water molecule and hydrated H^+^ counterion self-diffusion coefficient dependence on the diffusion time in the acidic form of the F-4CF membrane; relative humidity is 95%. Adapted from [24,38].

First of all, the results on the water and cation self-diffusion humidity dependences should be considered. The self-diffusion coefficients of water at different humidities are shown in Figure 23 for acid forms and Li^+^, Na^+^, and Cs^+^ ionic forms of sulfo MF-4SC and carboxylic F-4CF perfluorinated membranes. At the same λ self-diffusion coefficients of water in membrane MF-4SC and F-4CF salt forms are close to each other. In the Cs^+^ ionic form, the water self-diffusion coefficients are higher compared to those for the Li^+^ and Na^+^ ionic forms. In the acid ionic form, the self-diffusion coefficient in the sulfo-containing membrane is two to three orders of magnitude higher than that in the carboxylic membrane (curve 1 and curve 5, Figure 23). The restriction diffusion (decreasing self-diffusion coefficient with increasing diffusion time) occurs and the size of the restriction region is about 1 µm (Figure 24). It may be evidence for internal hydrogen bond formation between carboxyl groups.

**Figure 25:**
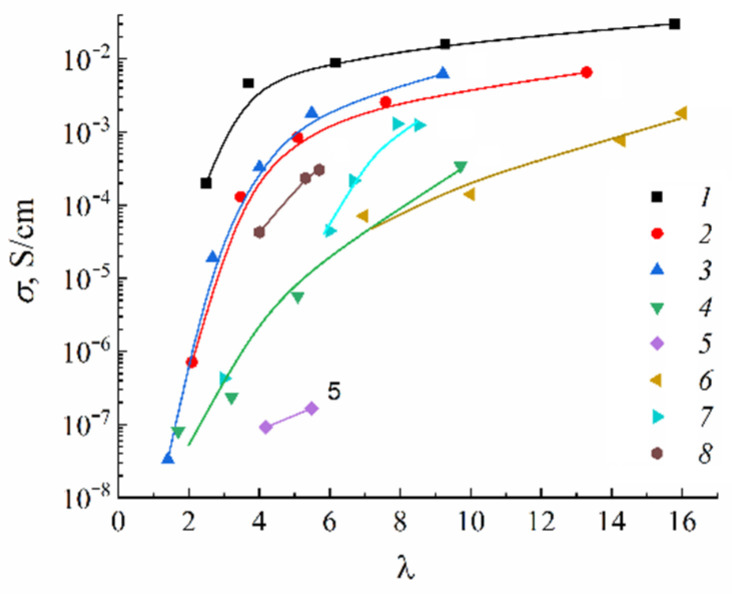
Conductivity dependence on the moisture content for sulfonate cation-exchange resin MF-4SC (1–4) and carboxylic F-4CF (5–8) perfluorinated membranes in various ionic forms. Membrane ionic forms: (1, 5) H^+^, (2, 6) Li^+^, (3, 7) Na^+^, (4, 8) Cs^+^. Adapted from [24,38].

The ionic conductivity humidity dependences are quite similar to the water self-diffusion coefficient temperature dependences (Figure 25).

In Table 5, the experimental ionic conductivities are compared with the conductivities calculated from Equation (7). These values are very close to each other, indicating the correlation character of water and cation translational mobility. The better agreement of NMR diffusion and conductivity data was obtained for Li^+^, Na^+^, and Cs^+^ cations, whose self-diffusion coefficients were measured.

As shown in Table 6, the calculated conductivities are slightly higher compared to the measured values. There is a principal reason for this difference. In the PFG NMR experiment, all cation translational motions are fixed, which is not obligatory, and accompanied by transfer along an applied electric field, such as it takes place in conductivity measurements. It should be expected that the agreement will be better in an electrophoretic NMR experiment with the application of an external electric field [25]. As indicated in Table 6, at relative humidity *RH* = 98% *λ* are 12, 10, and 4 for the Li^+^, Na, and Cs^+^ ionic forms, respectively, and the cation self-diffusion coefficients have changed in the following sequence: Li^+^ ≈ Na^+^ > Cs^+^.

**Figure 26:**
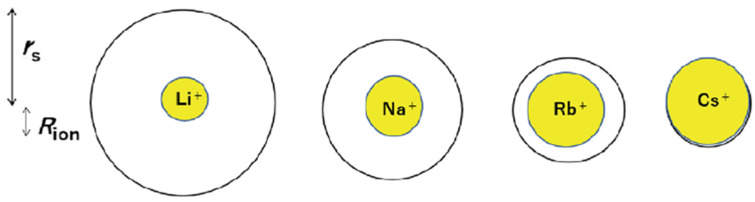
Relations between the dynamic ionic radius (*r*_s_) and static ionic radius (*R*_ion_) for the alkaline metal ions in aqueous solutions. Reprinted with permission from Ref. [112]. Copyright 2021 the Royal Society of Chemistry.

**Figure 27:**
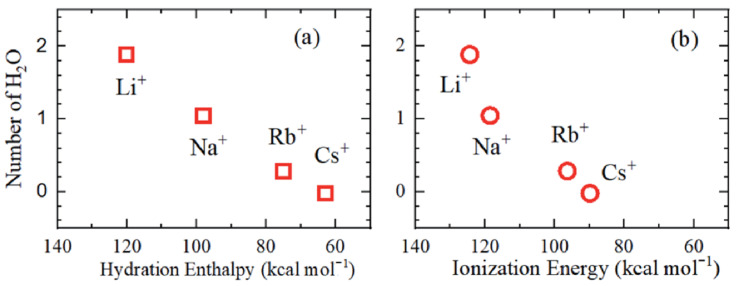
H_2_O numbers in the radial direction versus (**a**) hydration enthalpy and (**b**) ionization energy. Reprinted with permission from Ref. [112]. Copyright 2021 the Royal Society of Chemistry.

In the MSC membrane with a higher water capacity, the inverse sequence is observed, Li^+^ < Na^+^ < Cs^+^, which is of the same order as that in salt aqueous solutions. The cation self-diffusion activation energy increases from Li to Cs in the Nafion membrane, but is independent of the cation type in MSC and chloride aqueous solutions (Table 7). The sequence Li^+^ < Na^+^ < Cs^+^ indicates that the cation hydration radius, hydration enthalpy, and ionization energy decrease in this row (Figure 26 and Figure 27) [35,112].

The temperature dependences of the water and cation self-diffusion coefficients are approximated by the Arrhenius equation:

(8) $$D=D_{0}\cdot e^{\frac{E_{a}}{RT}},$$

where *D*_0_ is temperature independent, *R* is the gas constant, *T* is absolute temperature, and *E*_a_ is the self-diffusion activation energy.

The water molecule and cation self-diffusion activation energies increase with membrane dehydration, and the cation activation energy is higher than that of water (Table 8).

**Figure 28:**
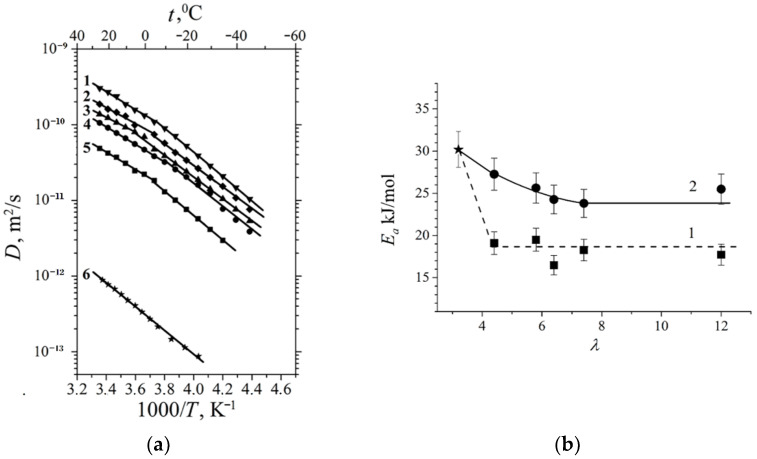
(**a**) Temperature dependences of the average H^+^ and water-self diffusion coefficients for the Nafion 117 membrane with different water contents: *λ* is 12 (1); *λ* is 7.4 (2); *λ* is 6.4 (3); *λ* is 5.8 (4); *λ* is 4.4 (5); and *λ* is 3.2 (6); (**b**) water self-diffusion activation energies in the high-temperature range (squares—curve 1) and low temperature range (circles—curve 2). Reprinted with permission from Ref. [41]. Copyright © 2022 Springer-Verlag GmbH Austria, part of Springer Nature.

At a high-water content, the slope of the self-diffusion and conductivity temperature dependences have changed and are characterized by two activation energies (high- and low-temperature ranges in Figure 28).

**Figure 29:**
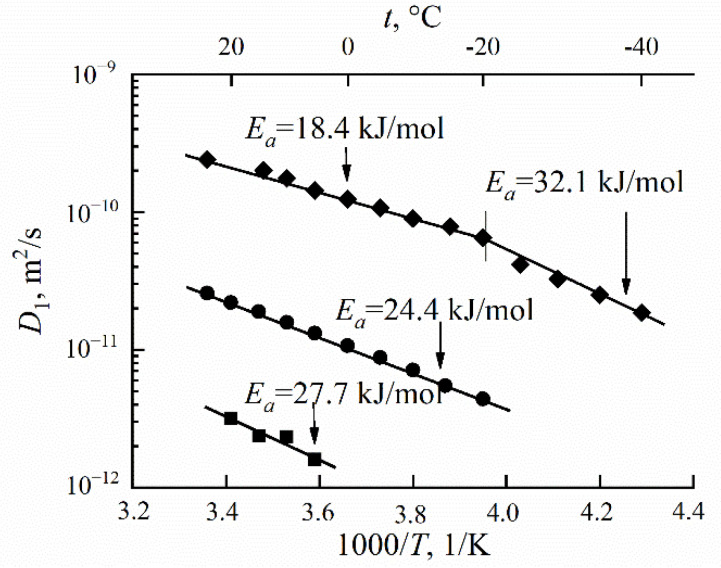
Temperature dependences of the self-diffusion coefficients *D* measured by PFG NMR at different water contents in the acid form of the MSC membrane: *λ* = 5.1 (1), 7 (2), 8.4 (3). Reprinted with permission from Ref. [34]. Copyright © 2022 Pleiades Publishing, Ltd.

**Figure 30:**
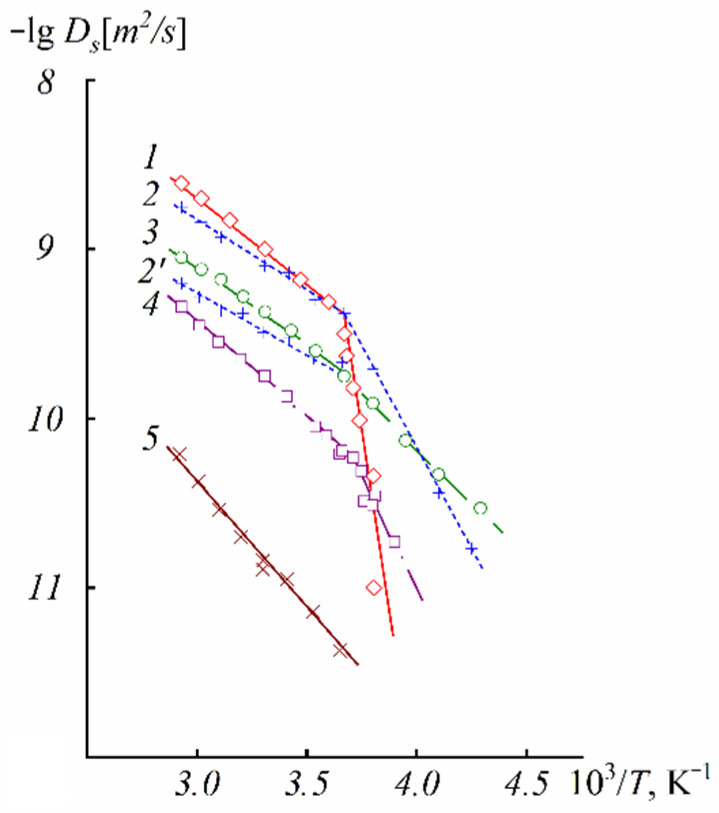
Temperature dependences of water self-diffusion coefficients at different water contents in the Ag^+^ ionic form of CU-23: 1, *λ* = 13.9; 4, *λ* = 4.1; and in H^+^ ionic form of the MC-44 membrane: 2,2′, *λ* = 15.1; 3, *λ* = 8.3; 5, *λ* = 1.4. Adapted from [33].

**Figure 31:**
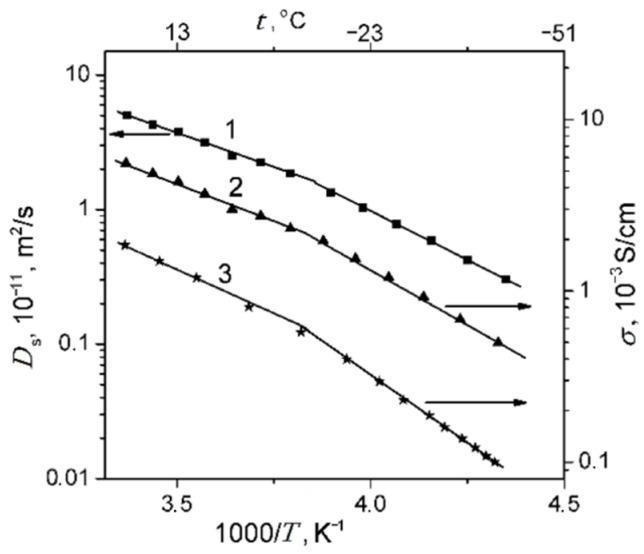
Temperature dependences of the self-diffusion coefficients (curve 1) and proton conductivity calculated from Equation (7) (curve 2), *λ* = 4.4. Curve 3: experimental temperature dependence of the conductivity for *λ* = 3.7. Adapted from [25,106].

At a low water content (*λ* ≤ *h*), the slope of the temperature curve does not change in the whole temperature range (curve 6, Figure 28a,b, Figure 29 and Figure 30). The same peculiarity of the temperature dependence is observed for other types of membranes (Figure 29 and Figure 30) and for the ionic conductivity temperature dependence (Figure 31).

Usually, the change in the temperature curve slope is explained by frozen unbounded water, which forms a separate ice phase. This is equivalent to decreasing hydrated water content *λ* and increasing activation energy. As shown in Figure 28a, the slope change is observed at lower *λ* compared to the value when water is still mobile below 0 °C. From our point of view, the reason for this rupture is the association of water molecules at the freezing temperature. The associated molecule self-diffusion activation energy is higher in the low-temperature range compared to bulk-like molecules at high temperatures. At a low humidity, all water molecules are fixed and the hydrogen bond network and activation energy are the same in the whole temperature range.

**Figure 32:**
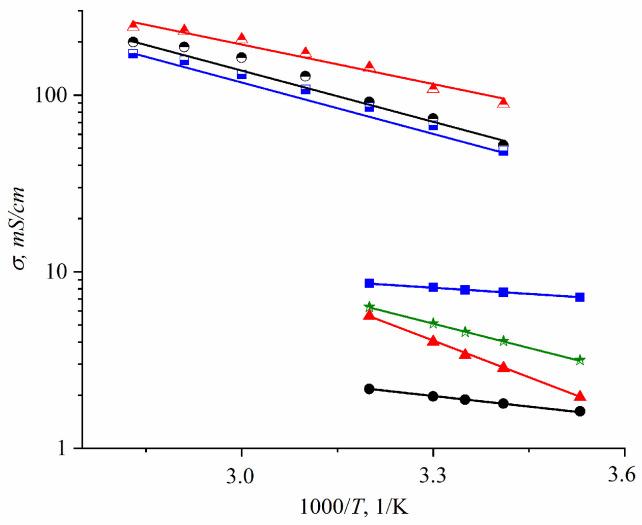
Temperature dependences of the experimental *σ*_exp_ (1–4) and calculated *σ*_calc_ (2′–4′) ionic conductivities in the H^+^ (1), Li^+^ (2) and (2′), Na^+^ (3) and (3′), Cs^+^ (4) and (4′) ionic forms of the MSC membrane at *RH* = 98%. Reprinted with permission from Ref. [35]. Copyright 2020 MDPI.

In cation-exchange membranes based on polyethylene and sulfonated grafted polystyrene (MSC), the experimental ionic conductivities are an order of magnitude lower compared to the values calculated from Equation (7) (Figure 32) [35].

This distinction is typical of membranes based on sulfonated polystyrene.

As mentioned above for these types of ion exchangers, the cluster-channel nanostructural model is suitable for the ion and molecular transfer. A version of this model, similar to the Gierke model, is shown in Figure 2. The pulsed field gradient NMR-measured self-diffusion coefficient is an average value of particles moving rapidly in clusters and slowly in channels, whereas the ionic conductivity is limited by slow passing through narrow channels.

**Figure 33:**
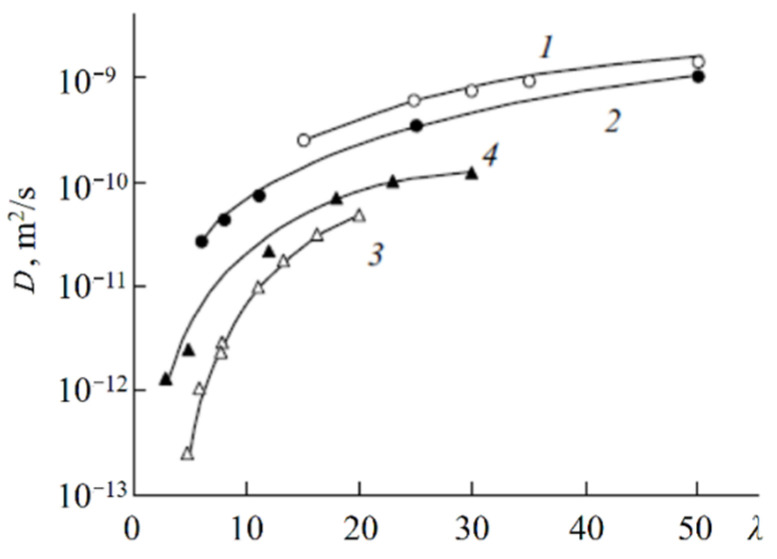
Diffusion coefficients of lithium cations in the system of lithium salt–disulfophthalic acid–water (1 is terephthalic PA-1 in Figure 34; 2 is isophthalic PA-2 in Figure 34) as a function of the moisture content in macroporous sulfonate cation-exchange CU-23 (4) and perfluorinated sulfonate cation-exchange membrane MF-4SC (3) [47]. Exchange capacity, mg-equiv/g: 0.86 for MF-4SC, 5 for macroporous cation exchanger CU-23, 2 for aromatic disulfo-containing polyamides [24].

The main disadvantage of all sulfo cation-exchange membranes is a low ion and water translational mobility and, consequently, conductivity at a low humidity. According to the ion transport mechanism discussed above, a continuous hydrogen bond network is necessary in order to accelerate cation and water molecule motions. This network is destroyed at a low humidity and, therefore, some fragments, which could form additional hydrogen bonds, should be involved in membranes. Inorganic dopants inserted into perfluorinated membranes increase the water mobility and ionic conductivity essentially [113]. Another way is a membrane synthesis with intermediate oxygen atoms (bridges), such as sulfo-containing aromatic polyamides. In Figure 33, the dependence of Li^+^ self-diffusion coefficients on λ are shown. Compared to perfluorinated membrane MF-4SC in polyamides, the lithium cation self-diffusion coefficient at a low water content is two to three orders of magnitude higher.

**Figure 34:**
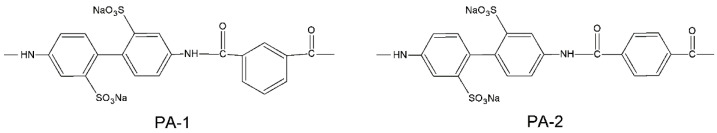
Chemical structure of sulfo-containing aromatic polyamides PA-1 and PA-2.

As mentioned above, the nanostructure of homogeneous ion exchangers based on sulfonated polystyrenes contains alternating cluster and channels. Diffusion in heterogeneous systems is more complicated. In [77] self-diffusion of Li^+^ cations and water molecules in macroporous sulfonic cation-exchange resin CU-23 was studied. Macropores fill up with water and contain sulfonate groups on the wall surface only. Therefore, the Li^+^ cation moves in the polymer phase through the system of cluster and channels. If a non-exchangeable electrolyte is involved in the macropore, the additional way for cation diffusion appears as a result of Li^+^ exchange between the polymer phase and an aqueous solution in a macropore. The dependences of self-diffusion coefficients on the water content *λ* and the concentration of external electrolyte LiCl and LiOH aqueous solutions were investigated.

**Figure 35:**
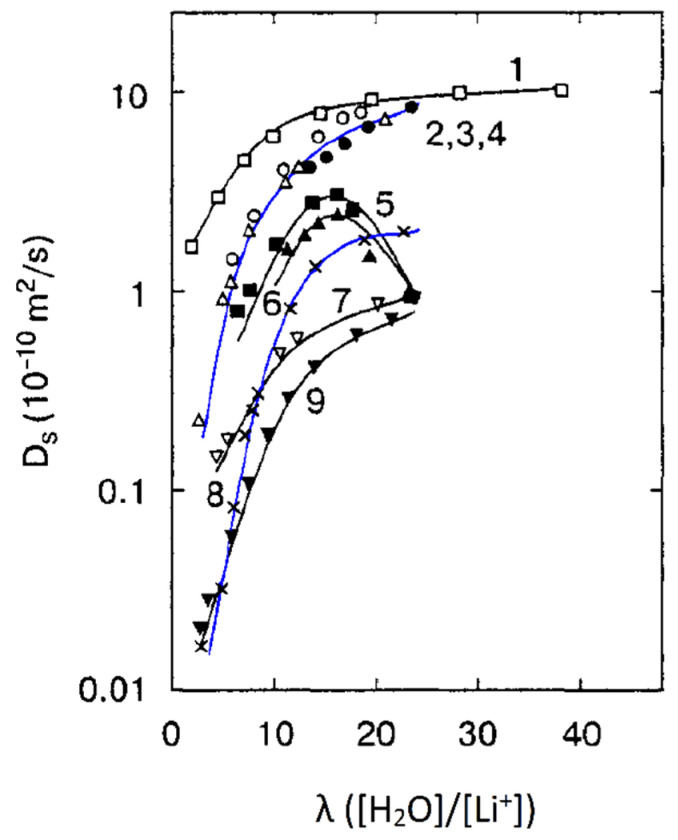
Dependence of the water self-diffusion coefficients (curves 2–4 and 8) and Li^+^ counterion self-diffusion coefficients (curves 1, 5–7 and 9) on *λ*, where *n* is the number of water molecules per one Li^+^ ion. Curve 1, Li^+^ in a LiCl aqueous solution; curve 2 and ∆, water in KU-23 without an external electrolyte; curve 3 and ○, water in KU-23 equilibrated with a LiCl aqueous solution; curve 4 and ●, water in KU-23 equilibrated with a LiOH aqueous solution; curve 5, Li^+^ in KU-23 equilibrated with a LiCl aqueous solution; curve 6, Li^+^ in KU-23 equilibrated with a LiOH aqueous solution; curve 7, Li^+^ in KU-23 without an external solution; curve 8, water in a Nafion-type membrane without an external solution: curve 9, Li^+^ in a Nafion-type membrane equilibrated with a LiCl aqueous solution. Adapted from [77].

**Figure 36:**
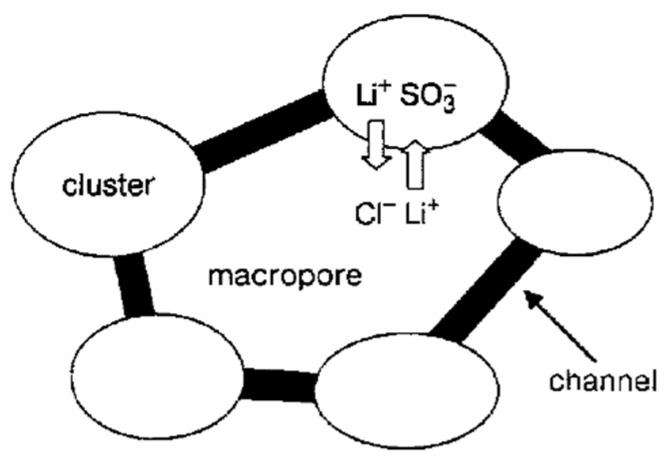
Structural model of CU-23 being in contact with a LiC1 aqueous solution. Adapted from [77].

With increasing solution concentration, some part of non-exchangeable sorption electrolytes LiCl or LiOH becomes involved with ion exchangers and the water content *λ* decreases. These *λ* values were calculated depending on the electrolyte solution concentration. Therefore, in Figure 35 (curve 6) the dependences of the water and lithium cation self-diffusion coefficients on *λ* are shown, where *λ* is the amount of water molecules per Li^+^ cation. Some rather interesting results were obtained. The water self-diffusion coefficients decreased with decreasing *λ*, and these dependences for the samples without an external electrolyte and for water contacting with the electrolytes were confirmed (curves 2, 3, and 4 in Figure 35). The lithium cation self-diffusion coefficient dependence in the samples without an external electrolyte solution is similar to that for water self-diffusion (curves 2, 3, 4, and 7, Figure 35). This result looks like the analogous dependence in the Nafion 117 membrane. In the samples equilibrated with electrolyte solutions, the Li^+^ self-diffusion coefficients reach a maximum. The maximum of Li+ self-diffusion coefficients is observed at *q*/*q*_o_ = 0.19 for LiCl and at *q*/*q*_o_ = 0.43 for LiOH aqueous solutions, where *q* is the solution concentration relative to *q*_o_ and *q*_o_ is the concentration of sulfonate groups (ion-exchange capacity), which is 5 meq/g. The water content *λ* was calculated at different concentrations of external electrolyte solutions. It turned out that the self-diffusion coefficients show maxima at the same *λ* regardless of the electrolyte type (curves 5, 6, Figure 35). These extreme dependences are explained in Figure 36 for a LiCl solution as an example. If the samples are swollen in pure water, the Li^+^ cation moves in the gel fraction of the ion exchanger through the cluster-channel network, which is similar to the cation transfer in homogeneous membranes. If the sample contacts with an electrolyte aqueous solution, the additional way for lithium cation translation appears as a result of Li^+^ exchange with Li^+^ cations sorbed in the macropore. An exchange rate increases with increasing concentration of the Li^+^ self-diffusion coefficient. With increasing electrolyte concentration, the *λ* value decreases, which is accompanied by decreasing of the self-diffusion coefficient. These two alternating factors are summarized and become a reason for maxima in the dependences.

## 5. Alcohol Molecule Self-Diffusion

The overwhelming majority of investigations of ion-exchange membrane transport properties is devoted to membranes swelling in water, but in some papers such organic solvents as saturated monoatomic alcohols, dimethyl sulfoxide, ethylene carbonate, and sulfolane are considered [45,50,52,53,55,99,101].

**Figure 37:**
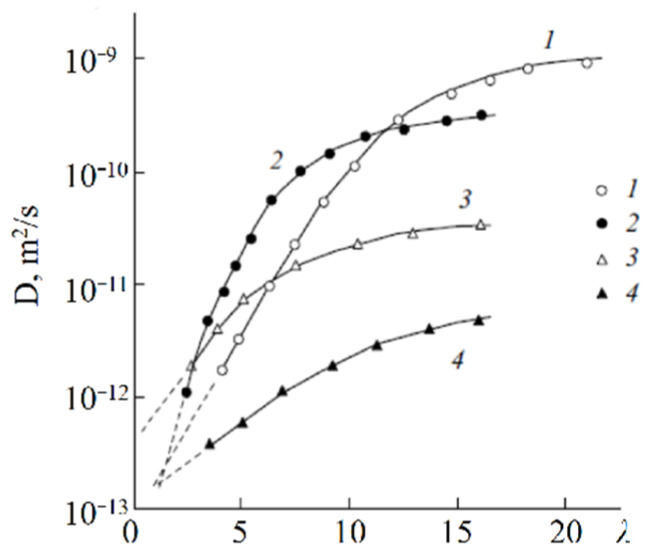
Self-diffusion coefficient dependences on the diffusant content of water (1), methanol (2), ethanol (3), and propanol (4) in the Li^+^-form of the perfluorinated membrane MF-4SC, λ is the number of diffusant molecules per sulfonate group (Adapted from [45]).

**Figure 38:**
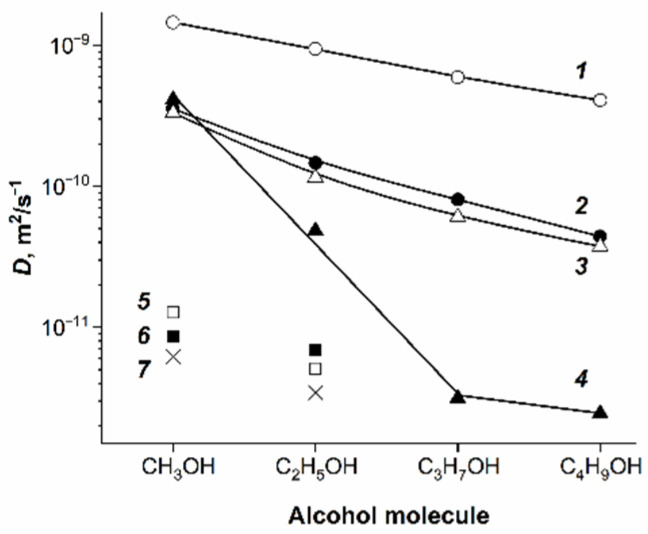
Diffusion coefficients of methanol, ethanol, propanol, and butanol in bulk liquids (1) and MF-4SC membranes in different ionic forms: Li^+^ (2); H^+^ (3); Na^+^ (4); Cs^+^ (5); Rb^+^ (6); K^+^ (7) (adapted from [45]). Bulk water self-diffusion coefficient at 25 °C is 2.3 × 10^−9^ m^2^/s, bulk methanol self-diffusion coefficient at 25 °C is 2.4 × 10^−9^ m^2^/s.

Alcohol self-diffusion in Nafion attracts attention from the point of view of development of fuel cells. Let us review briefly the main peculiarities of diffusivity in the row of saturated monatomic alcohols. In Figure 37, the dependences of water and saturated monatomic alcohol self-diffusion coefficients on the diffusant contents in the Li^+^ ionic form of the MF-4SC membrane are shown. The shapes of the dependences are similar. At a high solvent content, the self-diffusion coefficient of water is higher compared with those of alcohol. However, at a low solvent content, the self-diffusion coefficient of water is lower than those for methanol and ethanol. Cations are solvated by alcohol molecules, which is confirmed by a strong influence of cation types on the self-diffusion coefficients as shown in Figure 38. The slowness of methanol translational mobility compared to water at a high solvent content indicates an interaction of alcohol molecules with the polymer matrix.

## 6. Self-Diffusion of Molecules and Anions in Anion Exchangers

**Figure 39:**
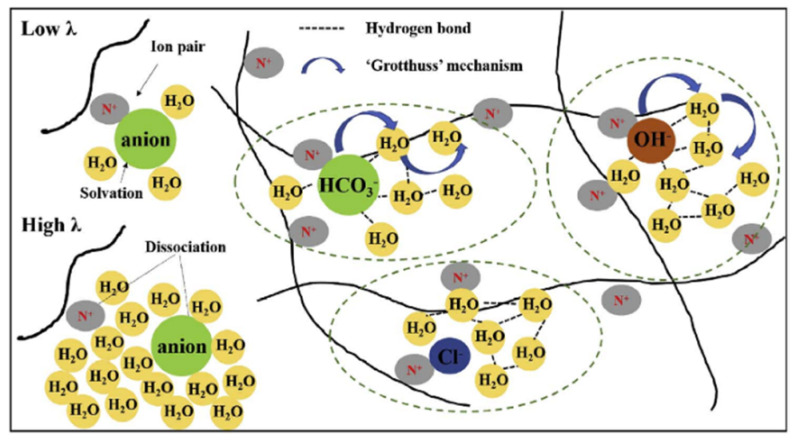
Schematic illustration of ion and water transport in Tokuyama A201. Reprinted with permission from Ref. [78]. Copyright © 2022 Published by Elsevier B.V.

**Figure 40:**
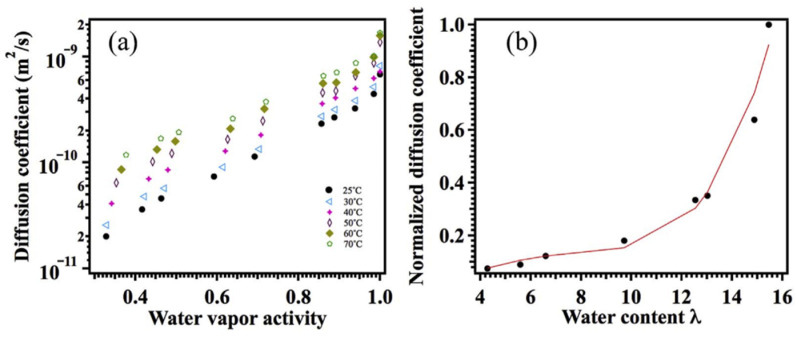
(**a**) Self-diffusion coefficient of water in the OH^−^ form of Tokuyama A201 as a function of the water vapor activity at 25 °C to 70 °C; (**b**) normalized water diffusion coefficient in the OH^−^ form membrane as a function of the water content *λ*. Reprinted with permission from Ref. [78]. Copyright © 2022 Published by Elsevier B.V.

In recent years, anion-exchange membrane fuel cells have attracted attention. As compared to cation-exchange membranes, hydroxide ions are exchanged through the membrane rather than protons. In Figure 39, the ion and water transport in a Tokuyama A201 membrane in the OH^−^, HCO_3_^−^, and Cl^−^ forms is shown [78]. There are similar water and ion mobilities in anion and cation-exchange membranes, especially in the OH^−^ and H^+^ forms of Tokuyama A201 and Nafion membranes, respectively. Anion transport is controlled by ion hydration, and the hydration energy decreases with an increasing anion radius. As shown in Figure 40, the water self-diffusion coefficient strongly depends on the membrane water content. Self-diffusion of water, methanol, and anions was investigated in detail on ^1^H, ^19^F, and ^13^C (^13^C labeled bicarbonate and methanol) nuclei in a polyethylene-b-poly(vinylbenzyl trimethyl ammonium) copolymer anion-exchange membrane [114].

The highest self-diffusion coefficient was observed for the F^−^ anion. The diffusion activation energy of water is lower than that for anions. The measured conductivity of the HCO_3_^−^ anion is higher compared to the value calculated from the Nernst–Einstein Equation (7), indicating the presence of OH^−^ ions increasing the measured conductivity.

Self-diffusion of water, ethanol, and F^−^ ions was studied by PFG NMR in Neosepta ACLE-5P, Selemion ASV, and MAP-1 membranes and the anion-exchange resin AV-17 was based on styrene/divinylbenzene (DVB) copolymer containing quaternary amine functional groups [46]. The samples in contact with water and an NH_4_F × HF aqueous solution were measured. The self-diffusion temperature dependences were approximated by the Arrhenius Equation (8). The average self-diffusion coefficients and activation energies are given in Table 9. The water self-diffusion activation energies are approximately equal for all anion exchangers and are about 6 kcal/mol (25 kJ/mol). The self-diffusion coefficients are low for the samples in an NH_4_F × HF aqueous solution, which explains the decreasing membrane water content compared to the samples swelling in pure water.

The water self-diffusion coefficients decrease if the crosslinking agent (DVB) amount increases, due to the water content decreasing. The self-diffusion coefficients measured by PFG NMR are higher compared to those obtained by the radioactive tracer technique (Table 10).

In contrast to water, ethanol is distributed non-homogeneously in anion exchangers. Two regions with different partial self-diffusion coefficients were observed. These coefficients *D*_i_ and relative parts *p*_i_ of absorbed ethanol molecules are shown in Table 11. The self-diffusion coefficient of the main part of ethanol (76%) is slightly lower than that of water, but about a quarter of ethanol molecules (24%) are moving more slowly by an order of magnitude. It may be supposed that alcohol molecules are situated not only near the charge groups but also interact with the polymer matrix.

The self-diffusion coefficients and activation energies of the F^−^ anion for the samples swelling in pure water and in an aqueous solution of NH_4_F × HF are given in Table 12. The self-diffusion coefficients are low, but the activation energies are higher for the fluorine anion than those for water molecules.

**Figure 41:**
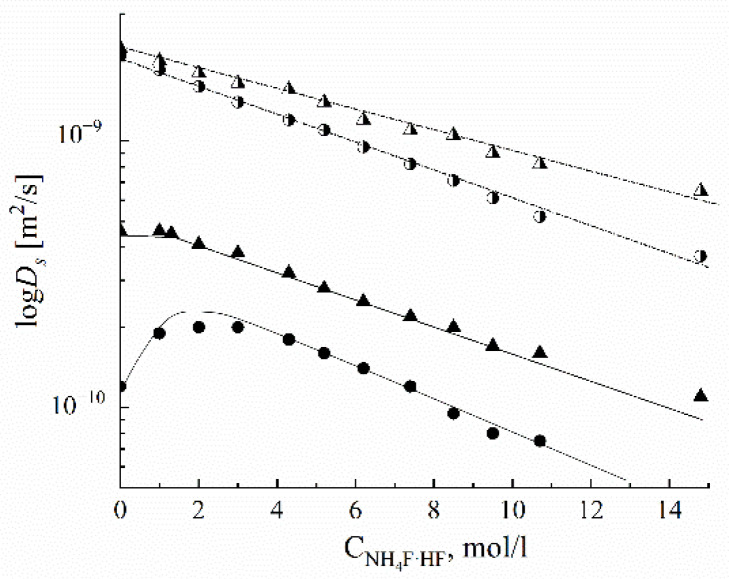
Dependencies of the F^−^ counterion and water self-diffusion coefficients on the concentration of the outer contacting electrolyte (aqueous solution of NH_4_F × HF), curves 1 and 2, respectively. Curves 1′ and 2′ are the F^−^ ion and water self-diffusion coefficients in an aqueous solution of NH_4_F × HF (*T* = 300 K).

The dependences of the water and F^−^ self-diffusion coefficients on the NH_4_F × HF aqueous solution concentration in the anion exchanger AV-17 are shown in Figure 41. In solution, the self-diffusion coefficients decrease with increasing concentration (decreasing water molecule amount per anion F^−^), as shown bycurves 1′ and 2′. The same tendency is observed for water in AV-17 (curve 2), but the fluorine anion self-diffusion coefficient shows a maximum (curve 1). The maximum originates from two opposite factors. On the one hand, decreasing water content and increasing concentration facilitates the contact of ionic pairs of F^−^ with anion groups, resulting in a decrease in the fluorine anion translational mobility. On the other hand, an increase in the amount of F^−^ anions due to a non-exchangeable adsorbed electrolyte results in an easier counterion transfer between ion-exchange sites (increasing translational mobility). Owing to these two factors acting in opposite directions, the maximum is observed. It is important to mention that the ionic conductivity also shows the maximum.

## 7. Inorganic Solid-State Proton Conductors

### 7.1. Solid-State Proton Conductors Based on CsHSO_4_-CsH_2_PO_4_

In most cases, proton transfer in proton conductors occurs in the system of hydrogen bonds of water molecules with a hydrated H^+^ cation. The parameters of proton transfer depend both on the structure of the crystal lattice and on the composition of the proton hydrate shell. Anhydrous hydrosulfates, hydroselenates, and dihydrophosphates of heavy alkaline metals are a separate group of proton electrolytes. The fast proton transfer in these systems is associated with a high symmetry of tetrahedral anions and, therefore, the possibility of its rotation [115]. The conductivity of these compounds is independent of an ambient humidity. The typical anhydrous proton conductors are CsHSO_4_ and CsH_2_PO_4_. CsHSO_4_ transits to a superionic phase with a high proton conductivity at a temperature of 141 °C, and remains up to a decomposition temperature of 210 °C. For CsH_2_PO_4_, a transition to the superionic phase is observed at 230–260 °C. The anhydrous proton conductors containing both SO_4_ and PO_4_ tetrahedra exhibit superionic conductivity over a wider temperature range compared to individual CsHSO_4_ and CsH_2_PO_4_, and a high proton conductivity can be retained until room temperature is reached.

### 7.2. CsHSO_4_ and Its Analogues

**Figure 42:**
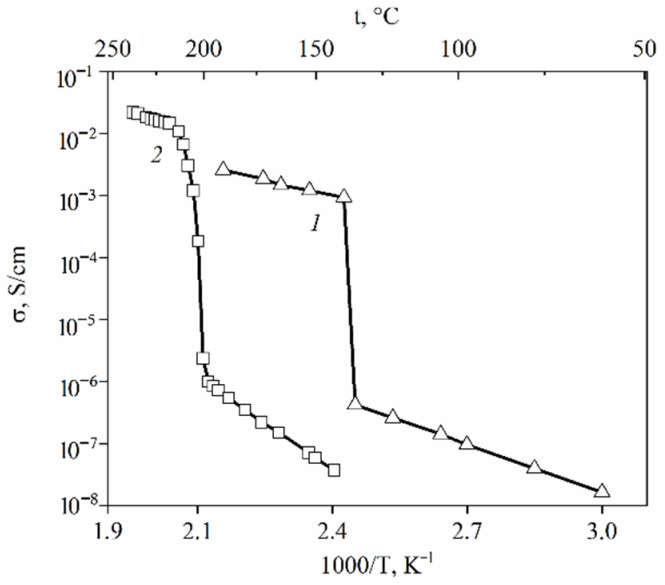
Temperature dependences of proton conductivity of CsHSO_4_ (curve 1 [116]) and CsH_2_PO_4_ (curve 2 [115]), adapted from [115,116].

The transition of CsHSO_4_ at 141 °C to the high-temperature superionic tetragonal phase I is of the greatest interest [116,117]. Upon transition to a highly conductive phase, the hydrogen bond (O…H-O) bends from 172° to 162°, the conductivity activation energy decreases from 0.50 eV to 0.30 eV, and the conductivity increases by three to four orders of magnitude to 10^–2^ S/cm (Table 13, Figure 42, curve 1 [116]). Phase transitions of cesium hydrosulfate were investigated by scattering neutron diffraction, inelastic neutron scattering, Raman spectroscopy, and periodic density functional theory calculations [118]. The authors put forward a new structure model that has no partial occupancies and provides specific O–H bond distances.

Composites of CsHSO_4_ (CHS) and heteropolycompounds xCsHSO_4_-(1-x)H_4_SiW_12_O_40_ (WSiA) with high proton conductivity have been obtained using the new liquid-phase shaking method. N,N-dimethylacetamide and zirconium beads were used as the dispersing mixing environment for the components [80]. X-ray diffraction analysis confirmed that such a treatment of the liquid phase induced an ion-exchange reaction between Cs^+^ in CHS and H^+^ in WSiA with the formation of new composite materials. The proton conductivity of these composites has been significantly improved compared to the starting materials, especially at low temperatures below the superconducting phase transition temperature of CHS. The 0.9CHS-0.1WSiA (mol%) composite showed the highest proton conductivity up to 2.42 × 10^−3^ S cm^−1^ at 170 °C. The measurements were carried out in a nitrogen atmosphere. The increased conductivity of anhydrous proton-conducting CHS-WSiA composites because of the reduction of hydrogen bond distances was also confirmed by ^1^H MAS NMR.

Inorganic solid proton-conducting composites prepared from phosphotungstic (WPA) heteropolyacid and inorganic solid acids MHSO_4_ (M = K and Cs-CHS and KHS, respectively) have demonstrated a high proton conductivity. The composition 90% (50KHS-50CHS)–10 mol% WPA showed the conductivity up to 4.9 × 10^−2^ S/cm at 160 °C. As shown by XRD, FT-Raman, ^1^H MAS-NMR, and HRTEM, the conductivity increase due to an amorphous surface of the WPA proton-conducting pathway formation has been shown to be the mechanism for increasing conductivity [81].

Recently, the electrotransport and thermal properties of Bu_4_NHSO_4_ were studied for the first time [119]. The compound slowly decomposes at a temperature of 260 °C. The proton conductivity of Bu_4_NHSO_4_ at 165–180 °C was about 10^–2^ S/cm. The conduction activation energy was 0.5 eV. The authors have explained proton conductivity as the binding of sulfate tetrahedra Bu_4_NHSO_4_ by hydrogen bonds.

### 7.3. CsH_2_PO_4_ and Its Analogues

The DSC data indicate two polymorphic transitions in CsH_2_PO_4_ at 149 °C (irreversible) and 230 °C (reversible-transformed to the cubic superionic phase) with decomposition temperatures above 250 °C [120]. It was shown [121] that the thermal effects at 220 °C and 255 °C correspond to the decomposition; first to Cs_2_H_2_P_2_O_7_ (175–225 °C) and then to CsPO_3_ (235–285 °C). These data were confirmed by the high-temperature X-ray studies and IR spectra. However, in recent years, most authors have proposed that at 230 °C CsH_2_PO_4_ transforms into a highly conductive cubic phase [122,123] with an increase by four orders of magnitude up to 2 × 10^−2^ S/cm (Figure 42, curve 2 [115]); thus, CsH_2_PO_4_ is a possible material for the production of fuel cells [124]. The conduction activation energy is 0.5–0.7 eV in the low-temperature range and 0.32–0.35 eV in the high-temperature range [123]. In a dry atmosphere, the conductivity of the high-temperature CsH_2_PO_4_ phase decreases due to the decomposition of the cubic phase. Starting from an RH humidity of 30% in an Ar atmosphere, the conductivity remains constant for several days at a fixed temperature [123].

The changing of hydrogen bonds was investigated by ^1^H and ^31^P MAS NMR in CsH_2_PO_4_ (CDP). Two lines in the ^1^H NMR spectra were observed below the temperature of the superproton phase transition corresponding to different networks of hydrogen bonds. The evolution of a line shape with temperature indicates hydrogen jumps in the structure of hydrogen bond networks. Proton conductivity was due to two types of hydrogen bonds, interchain and intrachain, in the environment of PO_4_ tetrahedra [82].

The MAS spectra of ^31^P NMR in a CsH_2_PO_4_ single crystal were analyzed in the temperature range from 260 to 420 K [83]. The ^31^P chemical shift drifts to a high field with increasing temperature. The linewidth (width at half-height) increases below 320 K and decreases above 320 K. The spin-lattice relaxation time *T*_1_ of ^31^P nuclei decreases linearly below 340 K and increases above 340 K. The spin-lattice mechanism is explained by the ^1^H-^31^P dipole–dipole interaction during the rotation of PO_4_ tetrahedra. The activation energies were 8.1 kJ/mol and *E*_a_ = 16.8 kJ/mol at the temperatures above and below 340 K, respectively.

Solid-state MAS NMR has been used to investigate changes in the dynamics of protons in acid salts MH_2_PO_4_ (M = K, Rb) [84]. The compounds exhibited a phase transition with an increase in proton conductivity over a range from room temperature to 110 °C. Double-quantum dipolar rebinding methods were used to quantify site-specific changes in the proton–proton dipolar interaction at different temperatures. The analysis made it possible to determine the apparent constants of the dipole–dipole bonds in KH_2_PO_4_ and RbH_2_PO_4_ as temperature changes. The authors of the work attribute the high proton conductivity to a noticeable weakening of the apparent dipole coupling. In a low-temperature monoclinic phase, RbH_2_PO_4_ has two chemically different proton environments, as shown in the ^1^H MAS NMR spectra.

The influence of small deviations from the stoichiometric Cs/P ratio in CsH_2_PO_4_ is observed [125]. To study the effect of small amounts of additions of H_3_PO_4_ or CsH_5_(PO4)_2_, measurements were carried out by impedance spectroscopy, powder X-ray diffraction, and IR spectroscopy. The methods of powder X-ray analysis and IR turned out to be insensitive to changes in the structure and in dynamic properties. The addition of CsH_5_(PO_4_)_2_ affects the temperature of the superionic phase transition and significantly increases the low-temperature conductivity.

Composite materials (1–*x*)CsH_2_PO_4_–*x*SnP_2_O_7_ (*x* = 0.01–0.8) were studied in [126]. The synthesis was carried out by the mechanical mixing of two components. X-ray diffraction analysis has shown that mechanical mixing of dispersed SnP_2_O_7_ with CsH_2_PO_4_ does not lead to the formation of new compounds. However, according to the data of impedance spectroscopy, the proton conductivity of the low-temperature range increases by one to three orders of magnitude. The X-ray powder diffraction and DSC data show a significant change in the structure. It is assumed that an increase in conductivity is determined by disordering in the structure of composite materials.

The structures and dynamics of the proton conductor CsH(PO_3_H) in the highly conductive phase were studied [85]. The existence of a phase transition at the temperature >137 °C is confirmed by NMR. The ^1^H, ^2^H, and ^31^P NMR isotropic rotation is associated with the fast local motion of protons. The ^17^O NMR spectra in the temperature range from 34 to 150 °C were analyzed. The activation energy calculated from the NMR data (0.27 eV) is noticeably lower than that obtained for the proton conductivity. In the high-temperature conducting phase, the proton conductivity in CsH(PO_3_H) is determined by the activation energy of the exchange between oxygen centers. It has been shown that several ^17^O NMR signals are observed even in the highly conductive phase. This behavior is not typical in the case of fast isotropic rotation of anions.

Proton conductor Cs_7_(H_4_PO_4_)(H_2_PO_4_)_8_ is obtained at elevated temperatures (above 130 °C) by the reaction of CsH_2_PO_4_ and CsH_5_(PO_4_)_2_ [86]. The conductive phase structure was determined by X-ray and ^31^P NMR methods. This compound has no ordered low-temperature phase. The conductivity of Cs_7_(H_4_PO_4_)(H_2_PO_4_)_8_ is *σ* = 5.8 × 10^−4^ S/cm at 140 °C. The structure of the CsSbF_3_(H_2_PO_4_) composite was characterized by X-ray diffraction.

Studies by ^1^H, ^19^F, and ^31^P MAS NMR, DSC, and impedance spectroscopy were carried out [87]. There is a transition to a high-temperature superionic phase (>390 K), as in CsH_2_PO_4_. This was illustrated by a variation of the MAS ^1^H, ^19^F, and ^31^P NMR spectra at different temperatures, which is due to the high ionic mobility of the fluorine and proton sublattices. A transition from a crystalline disordered phase to an amorphous phase has been observed at temperatures above 430 K. The diffusion of protons was determined by the NMR data. The ionic conductivity in CsSbF_3_(H_2_PO_4_) is 2.6 × 10^−4^ S/cm in the temperature range of 410–425 K. The conductivity decreases to 10^−5^ S/cm at the amorphous phase (at temperatures of 435–445 K).

Cs_3_(H_1.5_PO_4_)∙2H_2_O and Cs_3_(H_1.5_PO_4_)_2_ were obtained from aqueous solutions of Cs_4_P_2_O_7_∙4H_2_O and CsPO_3_ [127]. The structure of the single crystal was determined by X-ray diffraction at –173 °C.

Some composites based on CsH_2_PO_4_ pass into a highly conductive phase at the temperatures below 230 °C. In some of them, when the temperature is lowered to room temperature, a high proton conductivity is maintained [128].

### 7.4. Composite System Based on CsHSO_4_-CsH_2_PO_4_

The system of mixed solid proton electrolytes CsHSO_4_-CsH_2_PO_4_ with different ratios of components was studied intensively. At the moment, in addition to the initial components CsHSO_4_ and CsH_2_PO_4_, mixed composite materials of various compositions are known; for example, β-Cs_3_(HSO_4_)_2.5_(H_2_PO_4_)_0.5_, α-Cs_3_(HSO_4_)_2_(H_2_PO_4_), Cs_5_(HSO_4_)_3_(H_2_PO_4_)_2_, and Cs_2_(HSO_4_)(H_2_PO_4_) [129]. Data on phase transition temperatures *t_pt_*, decomposition/melt *t_dec._*, activation energy of conductivity *E_act._*, and values of proton conductivity *σ* are presented in Table 13. Their crystal structure and ionic conductivity have been studied. It was shown that all of them are protonic solid electrolytes. The value and temperature range of superionic conductivity for all compounds in the CsHSO_4_-CsH_2_PO_4_ system are different. The problem of finding the optimal composition of the proton solid electrolyte in the CsHSO_4_-CsH_2_PO_4_ system still exists.

**Table 13:** Data of phase transition temperatures *t_pt_*, decomposition/melt *t_dec._*, conduction activation energy *E_act._* (low-temperature and high-temperature superionic phases), proton conductivity values *σ* (low-temperature and high-temperature superionic phases) in individual compounds based on CsHSO_4_-CsH_2_PO_4_.

Compounds	*t_pt_*,°C	*t_dec._*,°C	*E_act._*, eV		*σ*, S/cm		Ref.
			Low Temp.	High Temp.	Low Temp.	High Temp.	
CsHSO_4_	141	203	0.5	0.3	10^−7^–10^−6^	10^−3^–10^−2^	[115,116,117]
CsH_2_PO_4_	230 *	250	0.6	0.33	10^−6^–10^−5^	>10^−2^ (up to 6·10^−2^)	[115,122,125]
β-Cs_3_(HSO_4_)_2.5_(H_2_PO_4_)_0.5_	126 **	175	0.7	0.37	<10^−5^	>6·10^−3^	[129]
α-Cs_3_(HSO_4_)_2_(H_2_PO_4_)	105 **	148	0.9	0.45	<10^−6^	>6·10^−3^	[129]
Cs_5_(HSO_4_)_3_(H_2_PO_4_)_2_	94 ***	180	0.8	0.38	<10^−6^	>4·10^−3^	[129]
Cs_2_(HSO_4_)(H_2_PO_4_)	82 ***	185	0.9	0.37	<10^−6^	>3·10^−3^	[129]

* The superionic phase remains stable only at PH2O ≥ 30 mol.% of the partial pressure of water. ** Phase transition temperature during the first heating. During subsequent heating-cooling cycles, the phase transition temperature decreases. *** The high-temperature phase is maintained even at room temperature for a long time after the heating-cooling cycle.

The solid-state inorganic salt Cs_2_(HSO_4_)(H_2_PO_4_) has a high proton conductivity in the superionic phase and, at the same time, it remains cooling, even to room temperature, for a long time [88]. In this work, we partially replaced Cs ions with ammonium ions. It was possible to include ammonium ions for up to 2.3% of Cs cations. Successful incorporation of ammonium ions is confirmed by the crystal structure measured by powder X-ray diffraction and the phase transition measured by thermal analysis. Evidence for the ^31^P MAS NMR structure is also presented. The paper presents the ^1^H and ^133^Cs MAS NMR and two-dimensional ^1^H{^31^P} correlation spectra (REDOR). The MAS ^1^H and ^133^Cs NMR spectra show that the inclusion of ammonium ions leads to an increase in structural disorder.

Recently, studies have appeared on the properties of the quaternary systems CsHSO_4_–CsH_2_PO_4_–NH_4_H_2_PO_4_–H_2_O. The phase composition and phase equilibria were studied. The conditions for obtaining a large list of superprotonic crystals were determined [130,131,132].

In the mixed composite material CsHSO_4_-Cs_3_(HSO_4_)_2_(H_2_PO_4_), the ratio was 1:1 mol/mol. The self-diffusion coefficients were determined by the pulsed field gradient (PFG) NMR [89]. The NMR-measured self-diffusion coefficients were compared with the diffusion coefficients calculated from the proton conductivity on the basis of the Nernst—Einstein Equation (7).

**Figure 43:**
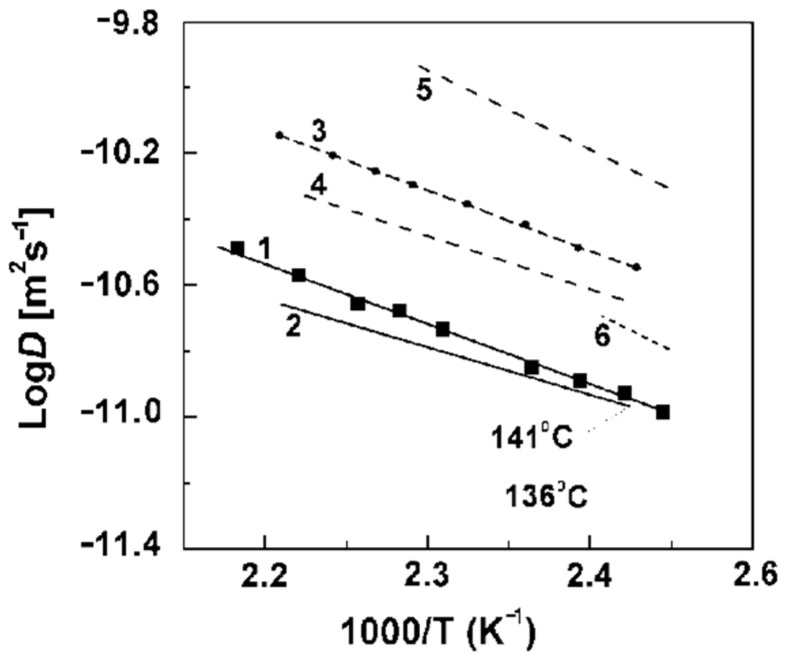
Temperature dependences of the diffusion coefficients of composite materials: 1, *D*_s_ obtained by PFG NMR for the composition CsHSO_4_-Cs_3_(HSO_4_)_2_(H_2_PO_4_) = 1:1; 2, *D*_s_ obtained by PFG NMR CsHSO_4_ [134]; 3, *D*_s_ calculated from Equation (7) for the composition CsHSO_4_-Cs_3_(HSO_4_)_2_(H_2_PO_4_) = 1:1; 4, *D*_s_ calculated from Equation (7) for CsHSO_4_; 5, *D*_s_ calculated from Equation (7) for β-Cs_3_(HSO_4_)_2.5_(H_2_PO_4_)_0.5_; 6, *D*_s_ calculated from Equation (7) for α-Cs_3_(HSO_4_)_2_(H_2_PO_4_) [89].

In Figure 43, the temperature dependences of the self-diffusion coefficients obtained by PFG NMR and calculated from Equation (7) are shown. According to the data of both NMR and impedance spectroscopy, the transition to the highly conductive phase of composite materials occurs at a temperature of 136–140 °C, which is consistent with the DSC data. The diffusion activation energy of the highly conductive CsHSO_4_ phase is 0.29 eV [133] and is in good agreement with the activation energy of the proton conductivity of CsHSO_4_ equal to 0.33 eV. The value of the activation energy of proton conductivity and diffusion coefficient obtained in this work for the composite material with the composition CsHSO_4_-Cs_3_(HSO_4_)_2_(H_2_PO_4_) = 1:1 is 0.37 eV. It should be noted that the values of the self-diffusion coefficients calculated from the conductivity exceed those obtained experimentally by PFG NMR. This may be due to the fact that the transfer of the hydrogen ion along the boundaries contributes to the conductivity, whereas diffusion in the volume only is observed by NMR. The second reason may be that the charge transfer of the proton by the Grotthuss mechanism is not included in the self-diffusion coefficient measured by NMR.

**Figure 44:**
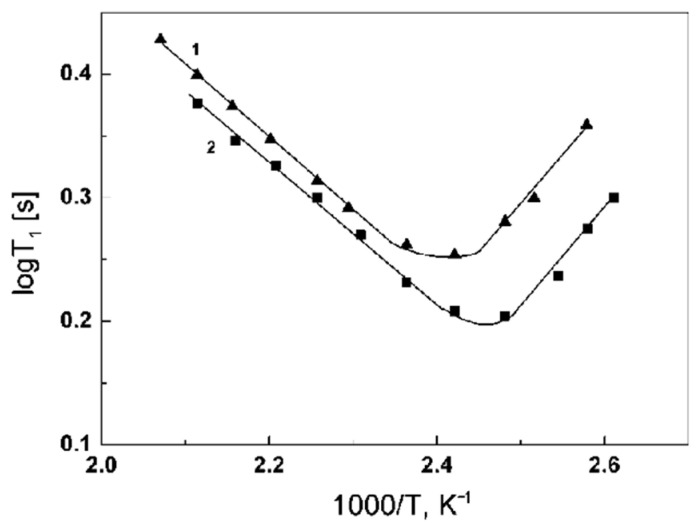
Temperature dependences of the ^31^P nuclei spin -lattice relaxation times *T*_1_ on (1) composite material Cs_2_(HSO_4_)(H_2_PO_4_)-CsH_2_PO_4_ = 2:1 and (2) α-Cs_3_(HSO_4_)_2_(H_2_PO_4_)-Cs_2_(HSO_4_)(H_2_PO_4_) = 1:1. Reprinted with permission Ref. [89]. Copyright © 2022 Springer-Verlag Wien.

**Figure 45:**
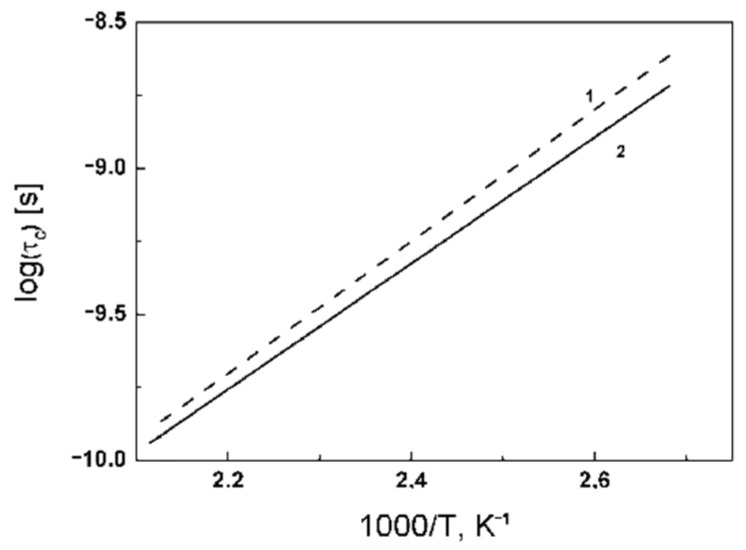
Temperature dependences of the correlation times *τ*_c_ of ^31^P nuclei: (1) composite material Cs_2_(HSO_4_)(H_2_PO_4_)-CsH_2_PO_4_ = 2:1 and (2) α-Cs_3_(HSO_4_)_2_(H_2_PO_4_)-Cs_2_(HSO_4_)(H_2_PO_4_) = 1:1. Reprinted with permission Ref. [89]. Copyright © 2022 Springer-Verlag Wien.

The relaxation times *T*_1_ on the ^31^P nuclei of the composite material CsHSO_4_:Cs_3_(HSO_4_)_2_(H_2_PO_4_) = 1:1 were analyzed. It is assumed that the proton transfer in the compounds and composite materials of the CsHSO_4_-CsH_2_PO_4_ system occurs due to the rapid rotation of the tetrahedra of the AO_4_ anion (A = S or P). The phosphorus atoms in the structure are firmly fixed and do not change their positions. Phosphorus in PO_4_ tetrahedra is surrounded by oxygen atoms, which have no magnetic moment. Cesium atoms are located far enough from phosphorus atoms (>4 Å). In this regard, the change in the magnetic moment of phosphorus nuclei in the tetrahedra will be affected only by changes in the positions of hydrogen atoms during rotation of the tetrahedron, due to the magnetic dipole–dipole interaction (distance between ^31^P and ^1^H is about 2.5 Å). The study of spin-lattice relaxation of ^31^P nuclei made it possible to determine the rotation parameters of PO_4_ tetrahedra. The temperature dependences of the spin-lattice relaxation times *T*_1_ ^31^P were obtained for the high-temperature phases of composite materials Cs_2_(HSO_4_)(H_2_PO_4_)-CsH_2_PO_4_ = 2:1 and α-Cs_3_(HSO_4_)_2_(H_2_PO_4_)-Cs_2_(HSO_4_)(H_2_PO_4_) = 1:1 (Figure 44). The curves show a minimum of *T*_1_ at temperatures of 130 and 140 °C. The activation energies calculated from the *T*_1_ temperature dependences is 0.43 and 0.45 eV, which is in good agreement with the activation energies of proton conduction for these composite materials. The coincidence of the activation energies confirms the rotational mechanism of proton conductivity in this class of compounds. Rotation is the limiting stage. Knowing the activation energy of rotation of the tetrahedra and the correlation time at the minimum, it is possible to construct the dependence of the correlation times on temperature over the entire range of superionic conductivity (Figure 45). As can be seen, the correlation times for different compositions are approximately equal, which is not surprising, since the process of rotation of tetrahedra does not depend much on the far environment. The correlation times vary in the range from 5 × 10^−8^ to 10^−10^, which means that the frequency of an elementary jump that can transfer a proton from one position to the next is about 109 times per second. According to the Einstein Equation (1), the calculated value is 2.5 Å, which is in good agreement with the value obtained from the structural data (2.7 Å).

## 8. Conclusions

The nanostructure of transport channels, cation-anion interaction, cation hydration, and ionic and molecular translational mobility in different spatial scales were revealed by high-resolution, spin-relaxation, and pulsed field gradient heteronuclear NMR techniques. The results of NMR applications to ion-exchange resins and membranes, and composite materials based on cesium acid sulfates and phosphates were summarized. A perfluorinated sulfonic cation membrane Nafion and membranes based on sulfonated-grafted polystyrene were considered in detail. A comparison of the water and cation self-diffusion coefficients calculated from the local mobility with the macroscopic self-diffusion coefficient, measured by PFG NMR, indicates that transport nanochannels are rod-like structures in Nafion and cluster-channel types in sulfonate polystyrene membranes. The cation hydration numbers *h* were calculated. It was shown that the H^+^ counterion forms the hydroxonium H_5_O_2_^+^ ion at low humidity. The hydration numbers and cation hydration radii decrease with decreasing hydration enthalpy. At low humidity where *λ* ≤ *h* (*λ* is the amount of water molecules per sulfonate group or cation), a contact ionic pair cation—SO_3_^−^ group is formed. At high humidity where *λ* > *h*, separated ionic pairs are formed. On the basis of these views, the mechanism of Nafion selectivity for a Cs^+^ cation in an aqueous solution of mixed cesium and sodium salts was understood. It was shown that the DSC peaks, high self-diffusion, and ion conductivity activation energies at a temperature below 0 °C are due to the association of the water molecule rather than to the freezing of unbounded water. In Nafion membranes, the alkaline ion cation self-diffusion coefficients decrease in the sequence: Li^+^ ≈ Na^+^ > Cs^+^. In sulfonated polystyrene membranes, the cation self-diffusion coefficients increase in the following row: Li^+^ < Na^+^ < Cs^+^. The ion conductivities calculated from the cation self-diffusion coefficients agree well with the measured conductivities in Nafion. Sulfonated polystyrene membranes show an order of magnitude lower in the experimental conductivity compared with the calculated value. These results are in agreement with the transport channel nanostructural models indicated above. The self-diffusion coefficient of Li^+^ cation dependences on the concentration of external LiCl or LiOH aqueous solutions in sulfonic cation resin CU-23 show a maximum, which is explained by the presence of macropores. A self-diffusion investigation of methanol, ethanol, propanol, and butanol in the perfluorinated sulfonic cation-exchange membrane MF-4SC show a similar character to water molecules. In contrast to water molecules, alcohol molecules interact with the membrane hydrophobic polymer matrix. Only few papers are devoted to anion-exchange systems. Self-diffusion of water, ethanol, and F^−^ anions was studied by PFG NMR in Neosepta ACLE-5P, Selemion ASV, and MAP-1 membranes and the anion-exchange resin AV-17 based on styrene/divinylbenzene (DVB) copolymer and containing quaternary amine functional groups. Samples in contact with water and an NH_4_F × HF aqueous solution were measured. The fluorine anion self-diffusion coefficient in AV-17 shows a maximum in the NH_4_F × HF aqueous solution concentration dependences. This is explained by the competition of two factors: increasing F^−^ mobility with sorption of a non-exchangeable electrolyte that follows humidity decreasing, which results in fluorine anion self-diffusion coefficient reduction. The mechanism of proton mobility in inorganic solid-state proton conductors is discussed. In heteropolycompounds, a proton is moving through the hydrogen bond network in the same manner as in the acid form of sulfonated membranes. The properties of a special class of solid electrolytes in which proton transfer occurs without water molecules are discussed.

These solid acids are characterized by a phase transition to the superionic phase with a stepwise increase in proton conductivity. The mechanism of ionic transport to the superionic phase is associated with proton transfer due to the rapid rotation of PO_4_ and/or SO_4_ tetrahedra. The studies were carried out using NMR PFG and NMR relaxation methods. Spin-lattice relaxation on ^31^P nuclei confirms that the proton transfer occurs due to the rotation of PO_4_ groups.

## Figures and Tables

**Table 1:** Self-diffusion coefficients of water molecule and lithium cation calculated from the ^1^H and ^7^Li relaxation data (*D*^calc^) and experimentally measured (*D*^exp^) [24,25].

Amount of Water Molecules per One Sulfonate Group (*λ*)	*D*^calc^_H2O_, m^2^/s	*D*^exp^_H2O_, m^2^/s	*D*^calc^_Li+_, m^2^/s	*D*^exp^_Li+_, m^2^/s
4	5 × 10^−12^	4 × 10^−12^	2 × 10^−12^	1 × 10^−12^
20.5	3 × 10^−10^	2 × 10^−10^	4 × 10^−11^	3 × 10^−11^

**Table 2:** Hydration number *h* at different absolute amounts of water molecules per ionic site SO_3_^−^ (*λ*) in the acidic form of the Nafion 117 membrane. Reprinted with permission from Ref. [41]. Copyright © 2022 Springer-Verlag GmbH Austria, part of Springer Nature.

*λ*, [H_2_O]/[SO_3_H]	Hydration Number *h*
1.9 ± 0.4	1.4 ± 0.5
3.2 ± 0.4	2.4 ± 0.5
4.4 ± 0.4	3.0 ± 0.3
5.8 ± 0.4	3.5 ± 0.3
6.4 ± 0.4	4.1 ± 0.3
7.4 ± 0.4	3.4 ± 0.3
12.0 ± 0.4	3.9 ± 0.3
17.5 ± 0.4	4.5 ± 0.5

**Table 3:** Hydration numbers *h* of Li^+^ cation in the lithium form of the Nafion 117 membrane at different water contents *λ*. Reprinted with permission from Ref. [60]. Copyright © 2022 Elsevier B.V. All rights reserved.

***λ*, [H_2_O/SO_3_^−^]**	0.9	2.0	4.0	5.7	7.4	10.7	12
***h***	0.6 ± 0.3	1.2 ± 0.5	2.1 ± 0.5	2.6 ± 0.5	2.9 ± 0.5	4.2 ± 1.0	5.0 ± 1.0

**Table 4:** Hydration numbers *h*_0_, hydration heat, and a part of cleaved hydrogen bonds in the MF-4SC membrane (adapted from [36]).

Ionic Form	*h*_0_ in Membrane	Hydration Heat–∆*H*, kJ/mol	Part of Cleaved Hydrogen Bonds Compared to Bulk Water
H^+^	2.0 ± 0.2	1150	-
Li^+^	3.6 ± 0.7	566	0.60
Na^+^	4.3 ± 0.7	475	0.50
K^+^	3.5 ± 0.9	392	0.30
Rb^+^	3.3 ± 0.7	362	0.25
Cs^+^	3.0 ± 0.6	333	0.20
Mg^2+^	6.7 ± 1.5	2042	0.60
Ca^2+^	7.2 ± 1.2	1708	0.50
Ba^2+^	7.1 ± 1.2	1442	0.30

**Table 5:** Ionic conductivities calculated from the diffusion coefficients of water and Li^+^ cations and measured for MF-4SC and F-4CF membranes in different ionic forms at a relative humidity of 90% (Adapted from [38]).

Ionic Form	*σ*_MF-4SC_, S/cm		*σ*_MF-4CF_, S/cm	
	Experiment	Calculation	Experiment	Calculation
H^+^	2.8 × 10^−2^	4.3 × 10^−2^	1.5 × 10^−7^	1.5 × 10^−6^
Li^+^	6.2 × 10^−3^	1.1 × 10^−2^ (see ^a^)	1.0 × 10^−3^	4.3 × 10^−3^
		6.5 × 10^−3^ (see ^b^)	-	-
Na^+^	6.1 × 10^−3^	1.2 × 10^−2^	1.2 × 10^−3^	4.6 × 10^−3^
Cs^+^	3.4 × 10^−4^	8.3 × 10^−4^	3.7 × 10^−4^	7.2 × 10^−4^

The calculations were performed from the diffusion coefficients of MF-4SC, F-4CF [37]: ^a^ water molecules and ^b^ lithium cations.

**Table 6:** Water content *λ*, hydration number *h*, self-diffusion coefficients *D*, calculated conductivity *σ*_c_, measured conductivity *σ*_e_ of Li^+^, Na^+^, and Cs^+^ cations in Li^+^, Na^+^, and Cs^+^ Nafion 117 membrane ionic forms, *RH* = 98%, *t* = 20 °C. Reprinted with permission from Ref. [60]. Copyright © 2022 Elsevier B.V. All rights reserved.

Ionic Form	Water Amount per Sulfonated Group *λ*	Hydration Number *h*	Cation Self-Diffusion Coefficient *D*, m^2^/s	Calculated Ionic Conductivity *σ*_c_, S/cm	Measured Ionic Conductivity *σ*_e_, S/cm
Li^+^	12	5.0 ± 1.0	(1.5 ± 0.1) × 10^−10^	(1.6 ± 0.1) × 10^−2^	(1.3 ± 0.1) × 10^−2^
Na^+^	10	6.0 ± 1.0	(2.0 ± 0.3) × 10^−10^	(2.0 ± 0.3) × 10^−2^	(1.1 ± 0.1) × 10^−2^
Cs^+^	4	1.0 ± 0.2	(0.6 ± 0.2) × 10^−10^	(6.0 ± 0.2) × 10^−3^	(2.3 ± 0.3) × 10^−3^

**Table 7:** Moisture content *λ*, amount of water molecules per cation, self-diffusion coefficients *D* at 20 °C, self-diffusion activation energies *E*_a_ of Li^+^, Na^+^, and Cs^+^ cations in the Nafion membrane, MSC membrane at *RH* = 98%, and chloride aqueous solutions [35,60].

Membrane Type	Cation	Moisture Content *λ*, Amount of Water Molecules per Cation	Cation Self-Diffusion Coefficient at 20 °C *D*, m^2^/s	Cation Self-Diffusion Activation Energy *E*a, kJ/mol
Nafion	Li^+^	12	(1.5 ± 0.1) × 10^−10^	20.5 ± 1.0
	Na^+^	10	(2.1 ± 0.3) × 10^−10^	19.3 ± 1.5
	Cs^+^	4	(0.6 ± 0.2) × 10^−10^	24.8 ± 1.5
MSC [35]	Li^+^	24	3.7 × 10^−10^	17.6
	Na^+^	21	4.4 × 10^−10^	18.1
	Cs^+^	16	8.3 × 10^−10^	16.5
Chloride aqueous solution	Li^+^	24	(8.2 ± 0.3) × 10^−10^	17.1 ± 0.5
	Na^+^	21	(1.1 ± 0.2) × 10^−9^	18.3 ± 0.6
	Cs^+^	16	(1.7 ± 0.2) × 10^−9^	16.8 ± 0.6

**Table 8:** Activation energies of Li^+^ and water molecule self-diffusion in the Li^+^ ionic form of the Nafion membrane *E*_a_ at different water contents *λ*. Reprinted with permission from Ref. [60]. Copyright © 2022 Elsevier B.V. All rights reserved.

***λ*, [H_2_O/SO_3_^−^]**	0.9	2.0	4.0	5.7	7.4	10.7
***E*_a_ of Li^+^ cation self-diffusion, kJ/mol**	40.3 ± 2.0	38.4 ± 2.0	28.8 ± 2.0	25.0 ± 2.0	25 ± 2.0	27.8 ± 2.0
***E*_a_ of water molecule self-diffusion, kJ/mol**	-	28.8 ± 2.0	25.0 ± 2.0	23.0 ± 2.0	21.1 ± 2.0	19.2 ± 2.0

**Table 9:** Average water self-diffusion coefficients *D* at 293 K and self-diffusion activation energies in the anion-exchange resin AV-17 and in the membranes ASV and ACLE-5P in the F^−^ form equilibrated with water and an 8 mol/L aqueous solution of NH_4_F × HF.

Ion-Exchange Material	Equilibrated with Water		Equilibrated with an 8 mol/L Aqueous Solution of NH4F × HF	
	*D*, (m^2^/s)	*E*_a_, (kcal/mol)	*D*_s_, (m^2^/s)	*E*_a_, (kcal/mol)
AV-17	4.6 × 10^−10^	6	1.25 × 10^−10^	6.6
ASV	1.9 × 10^−10^	6.3	1.3 × 10^−10^	6.7
ACLE-5P	5.9 × 10^−10^	5.9	2.28 × 10^−10^	5.8

**Table 10:** Self-diffusion coefficients of water molecules in the Cl^−^ form in MAP-1 membranes equilibrated with water and comprising various contents of DVB.

DVB Content, wt.%	Water Content, mol H_2_O/Functional Group	Water Self-Diffusion Coefficients *D*_s_, m^2^/s	
		PFG NMR Method	Radioactive Indicator Method
2	13.5	6.0 × 10^−10^	1.5 × 10^−10^
10	11.0	3.7 × 10^−10^	-
20	5.2	1.8 × 10^−10^	3.0 × 10^−11^

**Table 11:** Self-diffusion coefficients of water and ethanol (*D*_i_) at 293 K and relative values *p*_i_ of the diffusant in different regions of the ion-exchange materials in the F^−^ form equilibrated with water or ethanol.

Ion-Exchange Material	Equilibrated with Water		Equilibrated with Ethanol	
	*D*_i_, m^2^/s	*p*_i_	*D*_i_, m^2^/s	*p*_i_
AV-17	4.6 × 10^−10^	1	2.6 × 10^−11^	0.24
			1.5 × 10^−10^	0.76
			1.2 × 10^−10 a^	
ASV	1.9 × 10^−10^	1	4 × 10^−11^	0.24
			3 × 10^−10^	0.76
			2.38 × 10^−10 a^	

^a^ *D*_av_ values.

**Table 12:** Self-diffusion coefficients *D* at 293 K and self-diffusion activation energies *E*_a_ of F^−^ ions in the AV-17 resin and in the membranes ACLE-5P and ASV equilibrated with water or an aqueous solution of NH_4_F × HF.

Ion-Exchange Material	Self-Diffusion Coefficient *D*, m^2^/s	Activation Energy *E*_a_, kcal/mol
AV-17	-	-
Contact with water	1.1 × 10^−10^	7.2
Contact with 3.3 mol/L NH4F × HF	2.0 × 10^−10^	6.2
Contact with 8.0 mol/L NH4F × HF	7.2 × 10^−11^	7.2
Neosepta ACLE-5P	-	-
Contact with water	7.0 × 10^−11^	7.0
Contact with 8.0 mol/L NH4F × HF	6.2 × 10^−11^	7.5
Selemion ASV	-	-
Contact with water	10^−10^	8.5
Contact with 8.0 mol/L NH4F × HF	9.5 × 10^−11^	9.0
Aqueous solution of 8.0 mol/L NH4F × HF	6.2 × 10^−10^	5.0
